# Intratumoral delivery of RIG-I agonist SLR14 induces robust antitumor responses

**DOI:** 10.1084/jem.20190801

**Published:** 2019-10-10

**Authors:** Xiaodong Jiang, Viswanathan Muthusamy, Olga Fedorova, Yong Kong, Daniel J. Kim, Marcus Bosenberg, Anna Marie Pyle, Akiko Iwasaki

**Affiliations:** 1 Department of Immunobiology, Yale University School of Medicine, New Haven, CT; 2 Yale Center for Precision Cancer Modeling, Yale University School of Medicine, New Haven, CT; 3 Department of Molecular, Cellular, and Developmental Biology, Yale University, New Haven, CT; 4 Department of Molecular Biophysics and Biochemistry, W.M. Keck Foundation Biotechnology Resource Laboratory, Yale University School of Medicine, New Haven, CT; 5 Department of Dermatology, Yale University School of Medicine, New Haven, CT; 6 Department of Chemistry, Yale University, New Haven, CT; 7 Howard Hughes Medical Institute, Chevy Chase, MD

## Abstract

Cytosolic nucleic acid–sensing pathways can be triggered to enhance immune response to cancer. In this study, we tested the antitumor activity of a unique RIG-I agonist, stem loop RNA (SLR) 14. In the immunogenic tumor models, we observed significant tumor growth delay and an extended survival in SLR14-treated mice. SLR14 also greatly improved antitumor efficacy of anti-PD1 antibody over single-agent treatment. SLR14 was mainly taken up by CD11b^+^ myeloid cells in the tumor microenvironment, and many genes associated with immune defense were significantly up-regulated after treatment, accompanied by increase in the number of CD8^+^ T lymphocytes, NK cells, and CD11b^+^ cells in SLR14-treated tumors. Strikingly, SLR14 dramatically inhibited nonimmunogenic B16 tumor growth, and the cured mice developed an immune memory. Furthermore, a systemic antitumor response was observed in both bilateral and tumor metastasis models. Collectively, our results demonstrate that SLR14 is a promising therapeutic RIG-I agonist for cancer treatment, either alone or in combination with existing immunotherapies.

## Abstract (precis)

The authors examined a specific RIG-I agonist, SLR14, as an antitumor agent in mice. Intratumoral administration of SLR14 induces robust and long-term antitumor responses against primary, distal, and metastatic tumor as a single agent and improves efficacy of anti-PD1 therapy.

## Graphical Abstract

**Figure:**
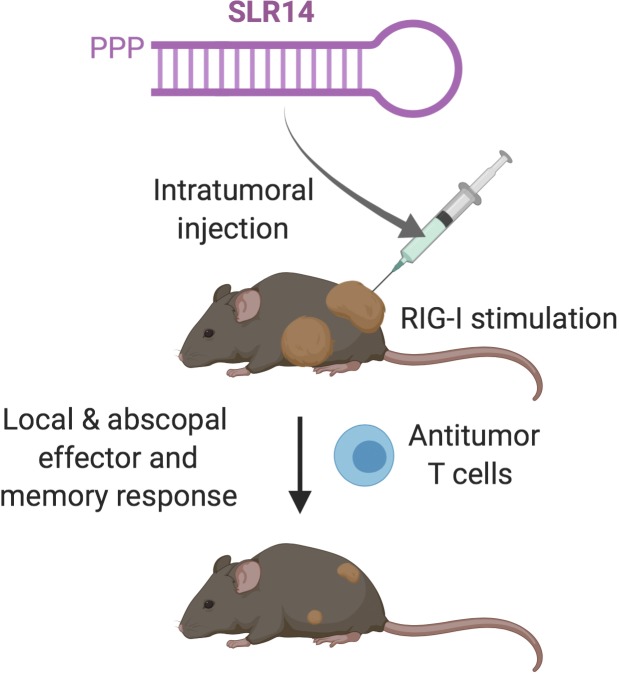

## Introduction

The innate immune system relies on pattern recognition receptors (PRRs) to sense invading microbes to initiate a rapid protective response. PRRs are activated by viral nucleic acids (NAs; Hlavata et al., 2018) or bacterial and fungal cell wall structures, collectively known as pathogen-associated molecular patterns (PAMPs; Medzhitov, 2007). Factors released by damaged tissues, or damage-associated molecular patterns, can also activate innate immune cells through engagement of PRRs and other receptors (Chen and Nuñez, 2010). The PRRs sensing microbial NAs are crucial for antimicrobial defense (Hlavata et al., 2018). These NA–sensing PRRs include (1) the endosomal TLR family (Majer et al., 2017); (2) the cytosolic DNA sensors cyclic GMP-AMP synthetase (cGAS) and AIM2 (Chen et al., 2016b); and (3) the cytosolic RNA sensors retinoic acid–inducible gene I (RIG-I)–like receptor family (Schlee, 2013). Once activated, these NA–sensing PRRs trigger multiple signaling cascades to induce the production of type I IFNs and proinflammatory cytokines, serving as the first line of defense against viral and microbial infections (Iwasaki and Medzhitov, 2010). Sensing NAs by PRRs has long been recognized as the critical mechanism for antiviral immunity. Interestingly, recent studies revealed that dying or damaged cancer cells could release NAs that can be recognized by cytosolic PRRs to induce antitumor immune response (Flood et al., 2019).

The cGAS-stimulator of interferon genes (STING) pathway is an important innate signaling pathway engaged upon recognition of cytosolic DNA. Dying or damaged cancer cell–derived DNA can act as a damage-associated molecular pattern sensed by cytosolic cGAS-STING machinery in tumor-associated CD8α^+^ dendritic cells (DCs), leading to the production of type I IFNs (Deng et al., 2014; Woo et al., 2014). Type I IFNs can exert their potent antitumor effects by enhancing CD8^+^ T cell priming and infiltration, as well as inducing cancer cell death through IFN–IFN-α/β receptor signaling (Zitvogel et al., 2015). cGAS stimulation in tumor cells provide ligands for STING in myeloid cells to promote NK cell activation and antitumor response (Marcus et al., 2018). As such, specific targeting of the cGAS-STING pathway presents a new opportunity for cancer immunotherapy. Antitumor effects of certain cGAMP derivatives and STING agonists (e.g., cyclic dinucleotides [CDNs]) have been reported in the mouse tumor models of skin, colon, breast, pancreatic, and B cell malignancies (Ng et al., 2018). cGAMP or STING agonists also show an enhanced antitumor response when combined with radiotherapy, chemotherapy, immune checkpoint inhibitors (PD-1 or CTLA4), or tumor vaccines (Deng et al., 2014; Fu et al., 2015; Wang et al., 2017a). The potential of the cGAS-STING pathway in the context of antitumor immunity shown by these preclinical studies has led to clinical evaluation of the antitumor efficacy of cGAS-STING agonists alone or in combination with other immunomodulatory agents (Iurescia et al., 2018). Yet, a canonical CDN, DMXAA, showed minimal effect on human STING (Conlon et al., 2013), suggesting that an optimal design for synthetic CDNs is required. Activation of cGAS-STING as a result of chromosomal instability in certain cancer types was found to promote epithelial-to-mesenchymal transition and metastasis (Bakhoum et al., 2018), tumor growth and increased regulatory T (T reg) cell infiltration, immunoregulatory cytokine IL-10, and indoleamine 2,3-dioxygenase enzyme (Ahn et al., 2015; Liang et al., 2015; Lemos et al., 2016). In addition, cGAS-STING signaling has been implicated in promoting tumor brain metastasis through gap junction–mediated cGAMP transfer from tumor cells to the astrocytes (Chen et al., 2016a). Such emerging evidence for cGAS-STING–mediated tumor promotion warrants further exploration of triggering other innate sensors for tumor therapy.

Cytosolic RNA sensor RIG-I, also known as DDX58 (Yoneyama et al., 2004), has been considered a key PRR participating in antiviral responses, especially against RNA viruses. RIG-I and melanoma differentiation-associated gene 5 (MDA5) share similar domains: they both have two N-terminal caspase activation and recruitment domains required for downstream signaling, a central DExD/H box RNA helicase domain with the capacity to hydrolyze ATP, and a C-terminal domain. However, RIG-I preferentially binds to short (>10-bp) dsRNAs that have blunt ends and a 5′-triphosphate (5′-ppp) moiety, while the MDA5 detects long dsRNAs (Kolakofsky et al., 2012; Fitzgerald et al., 2014; Mu et al., 2018). A signal proceeds from the ligand-bound RIG-I or MDA5 to the adaptor mitochondrial antiviral signaling protein and to IRF3 and NF-κB, which are activated and translocated into the nucleus to induce type I IFNs and other inflammatory antiviral molecules. Recent studies revealed that RIG-I and MDA5 might be temporally involved in the cytokine response; RIG-I appears to be involved in its initiation, while MDA5 may be more important for its persistence (Kasumba and Grandvaux, 2019).

Accumulating evidence has shown that activation of RIG-I/MDA5 signaling in cancers cells by RNA ligands (5′ppp RNA or oncolytic viruses) induces cancer cell apoptosis in a type I IFN-dependent or -independent manner (Poeck et al., 2008; Besch et al., 2009; Chiappinelli et al., 2015; Roulois et al., 2015; Yu et al., 2016), while depletion of RIG-I in human tumors confers resistance to ionizing radiation and many chemotherapy drugs (Ranoa et al., 2016). RIG-I signaling can also trigger the activation of DCs, NK cells, and subsequent CD8^+^ T cells to induce immunogenic death of cancer cells (Kübler et al., 2010; Ellermeier et al., 2013; Duewell et al., 2014). Interestingly, it was reported that RIG-I activation might inhibit tumor progression through regulating tumor hypoxia or gut microbiota (Engel et al., 2017; Zhu et al., 2017). These studies indicate the potential of RIG-I as a new therapeutic target for cancers. Thus far, tumor cell death induced by RIG-I activation has been reported in multiple types of human cancer cells, including pancreatic cancer, prostate cancer, head and neck squamous cell carcinoma, gastric adenocarcinoma, glioblastoma, and breast cancer (Wu et al., 2017; Elion et al., 2018). Several RIG-I–like receptor mimetics or agonists have been synthesized, and their antitumor effects are under investigation. A synthetic RIG-I–specific agonist, mimicking the structure of the influenza virus panhandle promoter (CBS-13-BPS), triggered significant tumor regression in a murine pancreatic tumor model (Lee et al., 2018). MK4621 (or RGT100), a synthetic RNA oligonucleotide activator of RIG-I, is being developed by Merck/Rigontec and currently is in phase 1 clinical trials for the treatment of advanced/metastatic solid tumor (NCT03739138, https://clinicaltrials.gov/ct2/show/NCT03739138). In addition, SB-9200 is reported to be a broad-spectrum antiviral innate sensor agonist that acts via the activation of the RIG-I and nucleotide-binding oligomerization domain 2 pathway (Jones et al., 2017); 5′ppp RNA with uridine-rich sequence with 99 nucleotides hairpin (M8) also specifically triggered RIG-I–mediated type I IFN response compared with other RIG-I aptamer and poly(I:C) (Chiang et al., 2015).

We previously designed and synthesized a set of polyphosphorylated RNAs with a stable tetraloop at one end of duplex RNA. RNA stem-loops as short as 10 or 14 bp (stem loop RNA 10 [SLR10] or SLR14) can induce a potent type I IFN response through RIG-I activation in vivo when intravenously delivered (Linehan et al., 2018). Given their small size and chemically defined composition, we believe SLRs represent a new class of therapeutic oligonucleotides with potential applicability as antitumor agents. To this end, in this study we evaluated the in vivo antitumor effect of SLR14 in different types of tumor models by intratumoral (i.t.) delivery.

## Results

### I.t. injection of SLR14 results in significant antitumor effects

**Figure 1.**
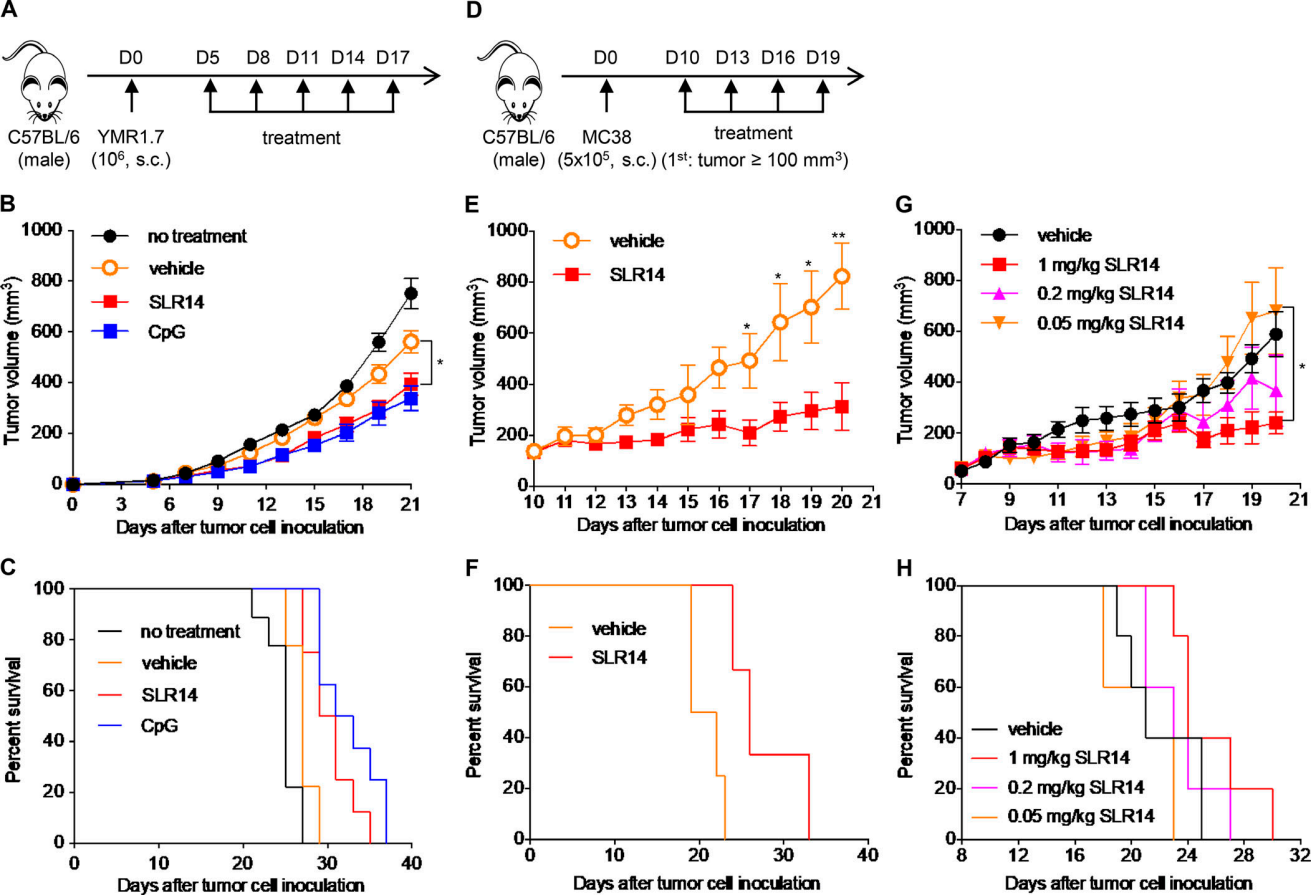
**I.t. injection of SLR14 results in significant antitumor effect. (A)** Subcutaneous YMR1.7 melanoma model was established in the right flank of C57BL/6J mice. At day 5 after injection, mice with similar tumor volumes were randomly divided into four groups (8–10 mice per group). The first group of mice was i.t. injected with 50 µl of 5% glucose mixed with 1 mg/kg (25 µg) SLR14 and 4 µl jetPEI (SLR14). The second group of mice was i.t. treated with 50 µl of 5% glucose containing 4 µl jetPEI (vehicle). The third group of mice was i.t. treated with 50 µl PBS containing 25 µg CpG. The fourth group of mice was i.t. treated with 50 µl of 5% glucose (no treatment). The treatment was performed every 3 d for a total of five doses. **(B)** Average tumor volume (error bars = SD) for each group of YMR1.7-bearing mice. **(C)** The survival curve of YMR1.7-bearing mice after treatment. **(D)** Subcutaneous MC38 colon cancer model was established at the right flank of C57BL/6J mice. When tumor volume reached ≥100 mm^3^ (day 10), the mice with similar tumor volumes were i.t. treated with 1 mg/kg (25 µg) SLR14 or vehicle (four to five mice per group). The treatment was performed every 3 d for a total of four doses. **(E)** Average tumor volume (error bars = SD) for each group of MC38-bearing mice. **(F)** The survival curve of MC38-bearing mice after treatment. **(G)** Average tumor volume (error bars = SD) of MC38-bearing mice receiving different doses of SLR14 (1, 0.2, or 0.05 mg/kg) or jetPEI (vehicle). Five mice per group. **(H)** The survival curve of MC38-bearing mice receiving different doses of SLR14. Multivariate analysis of variance and multiple *t* tests were used for statistical analysis. *, P < 0.05; **, P < 0.01. Results are representative of at least two independent experiments.

We first used subcutaneous YUMMER (Yale University mouse melanoma exposed to radiation; YMR) 1.7 melanoma mouse model to evaluate the antitumor effect of SLR14 in vivo (Fig. 1 A). The YMR1.7 line was generated by UVB irradiation of YUMM (YM) 1.7 murine melanoma cell line, which was derived from tumor arising in a *Braf*^V600E^, *Pten*^−/−^, and *Cdkn2a*^−/−^ mouse model of melanoma (Meeth et al., 2016). YMR1.7 cells carry high somatic mutation load and recruit a large number of tumor-infiltrating lymphocytes when injected in vivo (Wang et al., 2017b). We used jetPEI as the vehicle to deliver SLR14 transfection i.t. to focus immune activation to the tumor and its local environment. Both jetPEI and i.t. administration have been successfully used in other in vivo tumor studies (Li et al., 2016; Elion et al., 2018; Linehan et al., 2018). The mice treated with CpG1826 (CpG), jetPEI (vehicle), or water with 5% glucose (no treatment) were used as controls. After five doses of i.t. injection, a significant delay of tumor growth was observed in SLR14- or CpG-treated mice (Figs. 1 B and S1 A). These SLR14- or CpG-treated mice also displayed an improved survival course compared with vehicle-treated mice (Fig. 1 C). We observed that CpG had a slightly better treatment efficacy than SLR14. Although the tumors in vehicle-treated mice were slightly smaller than those in no-treatment mice, we did not observe any statistically significant difference between these two groups of mice. We also observed a similar antitumor activity of SLR14 in another immunogenic tumor model, MC38 colon cancer, even when the SLR14 injection was given at a late stage when the tumor mass reached 100 mm^3^ (Fig. 1, D and E; and Fig. S1 B). We further demonstrated that the antitumor effect of SLR14 was dose dependent (Fig. 1, G and H). Mice were euthanized according to our animal protocol criteria (tumor volume >1 cm^3^). Taken together, these results clearly demonstrate that SLR14, when delivered i.t., can induce a potent antitumor response against these immunogenic tumors.

### Combination treatment with SLR14 and anti-PD1 leads to better antitumor effects than single treatment

**Figure 2.**
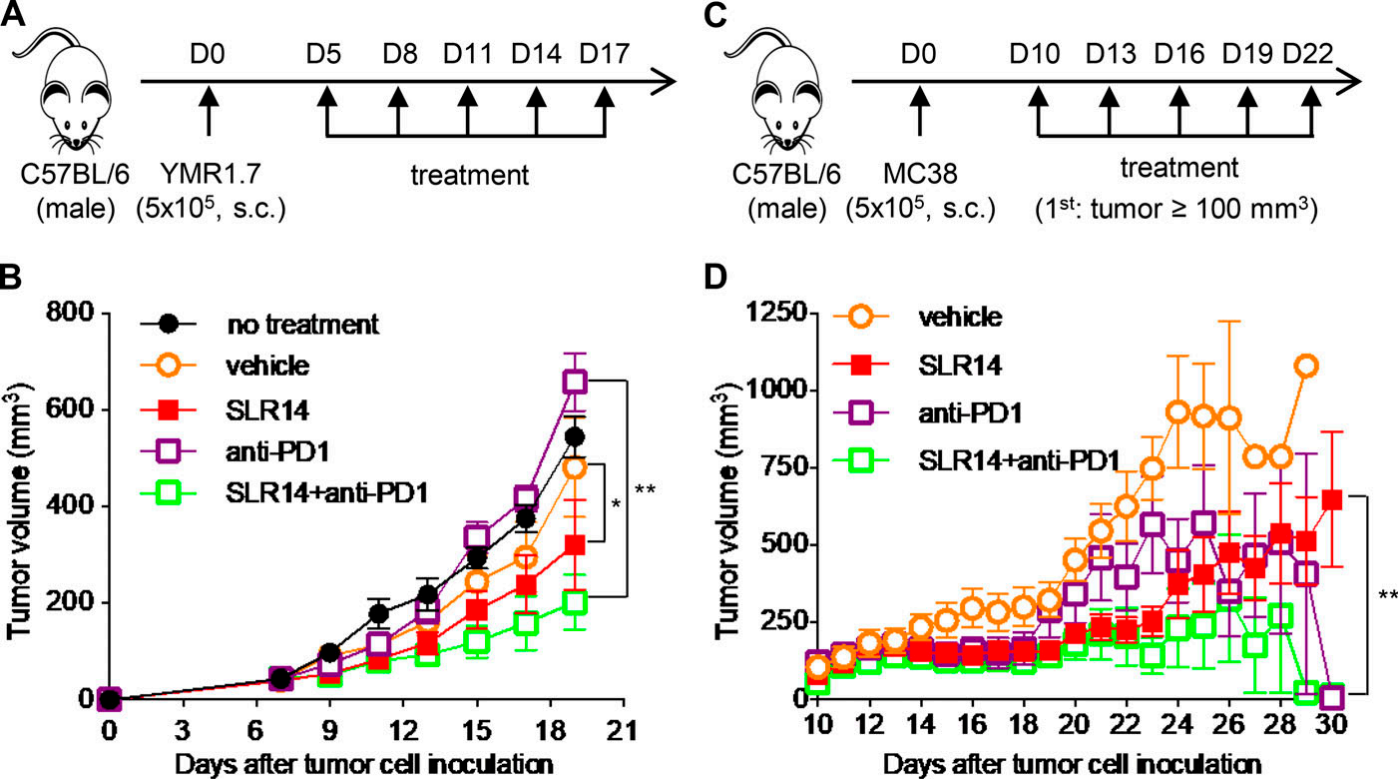
**Combination treatment with SLR14 and anti-PD1 leads to better antitumor effects than single treatment. (A)** Subcutaneous YMR1.7 melanoma model was established as described in Fig. 1 A. At day 5 after injection, the mice with similar tumor volumes were randomly divided into five groups (five mice per group) for i.t. treatment with vehicle, SLR14 (1 mg/kg) or no treatment, i.p. treatment with anti-PD1 (200 µg per mouse), or SLR14 (i.t.) plus anti-PD1 (i.p.). The treatment was performed every 3 d for a total of five doses. **(B)** Average tumor volume (error bars = SD) for each group of YMR1.7-bearing mice. **(C)** Subcutaneous MC38 colon cancer model was established as described in Fig. 1 D. When tumor volume reached ≥100 mm^3^ (day 10), the mice with similar tumor volumes were randomly divided into four groups (5–10 mice per group) for i.t. treatment with vehicle or SLR14 (1 mg/kg), i.p. treatment with anti-PD1 (5 µg per mouse), or SLR14 (i.t.) plus anti-PD1 (i.p.). The treatment was performed every 3 d for a total of five doses. **(D)** Average tumor volume of individual MC38-bearing mice in each group (error bars = SD). Multivariate analysis of variance was used for statistical analysis. *, P < 0.05; **, P < 0.01. Results are representative of two independent experiments.

As YMR1.7 clearance depends on T cells and is sensitive to immune checkpoint inhibitors including anti-CTLA4 and anti-PD1 (Wang et al., 2017b), we next examined if SLR14 and anti-PD1 combined treatment might improve the antitumor efficacy of single treatment. To this end, YMR1.7-bearing mice were generated as described in Fig. 1 A and treated with SLR14 (by i.t. injection), anti-PD1 (by i.p. injection), or SLR14 plus anti-PD1, for a total of five doses (Fig. 2 A). As expected, we again detected a significant delay of tumor growth in the mice treated with SLR14 alone. Although no significant benefit was detected in the mice treated with anti-PD1 as a single therapy, we observed a significant reduction in tumor growth in the mice with SLR14 and anti-PD1 combination treatment (Figs. 2 B and S2 A). We also tested combination treatment in the MC38 colon cancer model (Fig. 2 C). As MC38 is extremely sensitive to 200 µg anti-PD1 i.p. treatment based on our pilot experiment, we decided to examine whether SLR14 can synergize with a very low dose of anti-PD1 antibody (5 µg). As shown in Figs. 2 D and S2 B, tumor growth was remarkably impeded after SLR14 and anti-PD1 combination treatment, compared with single treatment. These findings indicate that SLR14 induces a synergistic antitumor effect when combined with immune checkpoint inhibitor anti-PD1 for YMR1.7 or MC38 treatment.

### SLR14 is mainly taken up by CD11b^+^ myeloid cells in the tumor microenvironment

**Figure 3.**
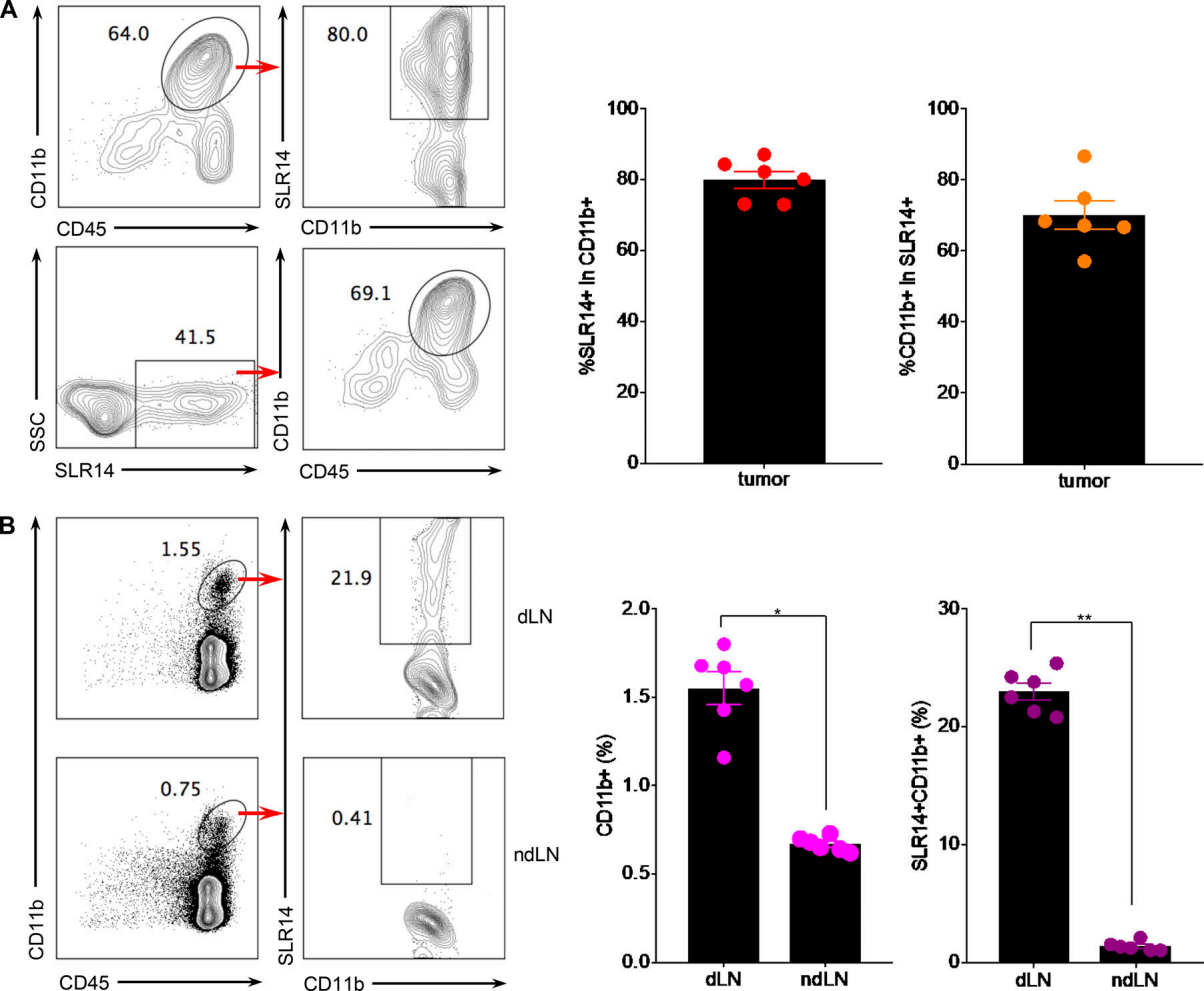
**SLR14 is mainly taken up by CD11b^+^ myeloid cells in the tumor microenvironment.** Subcutaneous YMR1.7 melanoma model was established in C57BL/6J mice (six mice) as described in Fig. 1 or 2. At day 12 after injection, the mice were i.t. treated with 50 µl of 5% glucose containing 1 mg/kg (25 µg) AF647-conjugated SLR14 and 4 µl jetPEI (SLR14). 24 h later, tumors were harvested and digested to make single-cell suspensions for flow cytometry analysis. **(A)** Top: The percentage of SLR14^+^ cells in total CD11b^+^ cells within tumor; bottom, the percentage of CD11b^+^ cells in total SLR14^+^ cells within tumor. Error bars = SD. **(B)** The percentage of CD11b^+^ cells in total CD45^+^ cells and the percentage of SLR14^+^ cells in total CD11b^+^ cells in dLN (top) and ndLN (bottom). Error bars = SD. Unpaired *t* test was used for statistical analysis. *, P < 0.05; **, P < 0.01. Results are representative of two independent experiments.

RIG-I is ubiquitously expressed in all cell types including cancer cells. Published studies (Poeck et al., 2008; Besch et al., 2009; Kübler et al., 2010; Ellermeier et al., 2013; Duewell et al., 2014; Chiappinelli et al., 2015; Roulois et al., 2015; Yu et al., 2016) have reported that RIG-I ligands could trigger RIG-I activation either in tumor cells, directly leading to cancer cell apoptosis, or in immune cells (e.g., DCs) to induce cancer immunogenic cell death. To determine whether SLR14, after i.t. injection, targets tumor cells or nontumor cells, we conjugated SLR14 with Alexa Flour (AF) 647 and i.t. injected AF647-SLR14 into YMR1.7 at day 12 after tumor cell injection. 24 h later, treated tumors were harvested, and SLR14^+^ cell populations were analyzed by flow cytometry. We found that ∼64% of the tumor-infiltrating leukocytes were CD11b^+^ cells. Strikingly, ∼80% CD11b^+^ tumor-infiltrating cells had incorporated SLR14. Of the total SLR14^+^ cells, we found that most of them (∼69.1%) were CD11b^+^ myeloid cells, and only a few were CD45^−^ (Fig. 3 A). Although we did not further analyze the nature of SLR14^+^CD45^−^ cells, this population likely includes some tumor cells, as well as other stromal cells in the environment. Next, we analyzed CD11b^+^SLR14^+^ cells in tumor-draining lymph node (dLN) or nondraining lymph node (ndLN) 24 h after i.t. injection of AF647-SLR14. Our results showed that ∼21.9% of CD11b^+^ cells in dLN had taken up SLR14, while no SLR14^+^CD11b^+^ cells were detected in ndLN (Fig. 3 B). Taken together, these results indicate that i.t. injected SLR14 is mainly taken up by CD11b^+^ tumor-infiltrating myeloid cells, and some SLR14^+^CD11b^+^ cells can be found in the dLN 1 d after the treatment.

### I.t. SLR14 delivery promotes immune activation and tumor infiltration of cytotoxic T lymphocytes as well as myeloid cells

**Figure 4.**
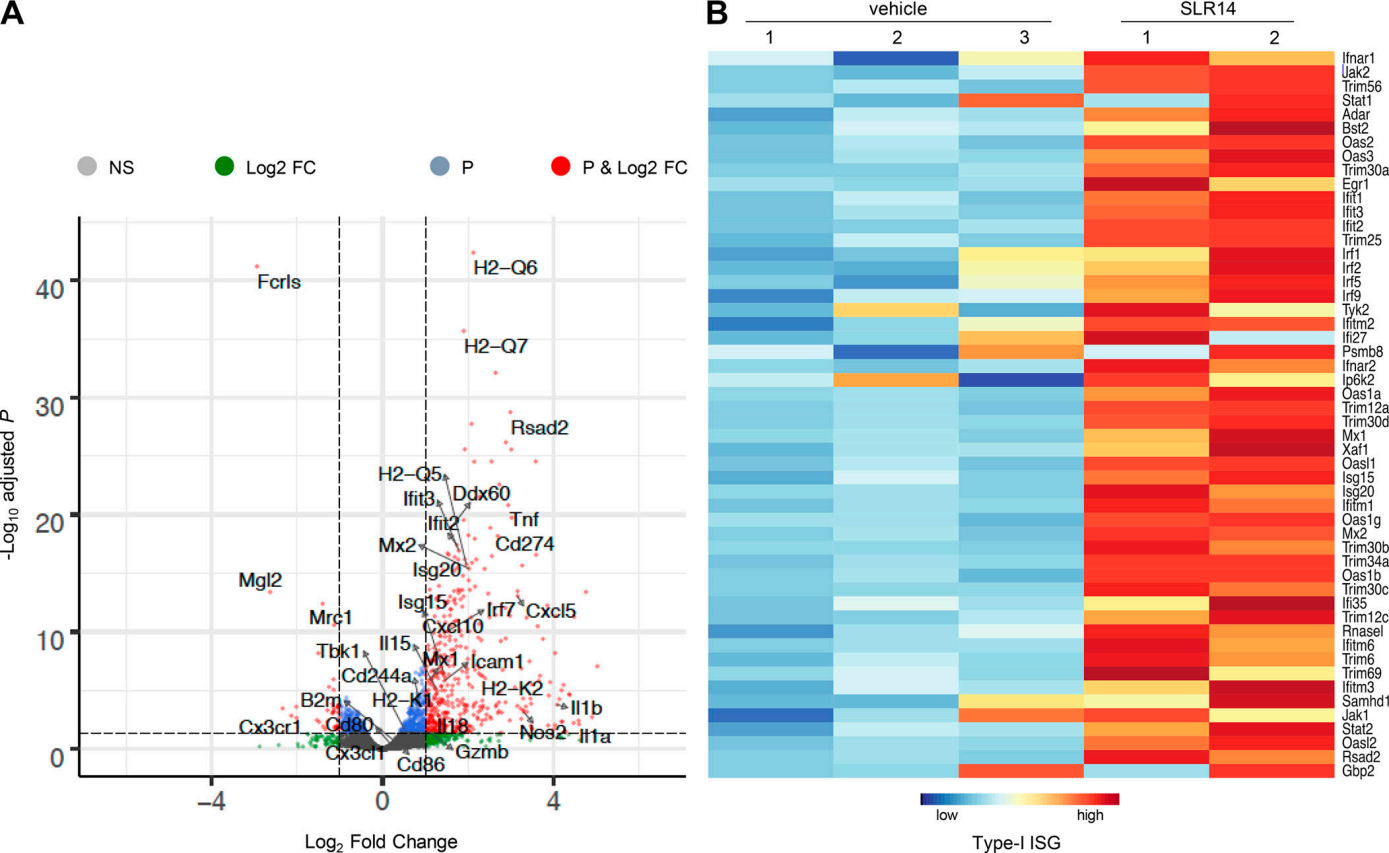
**Transcriptomic analysis of tumor i.t. treated with SLR14 versus vehicle.** Subcutaneous YMR1.7 melanoma model was established in C57BL/6J mice and i.t. treated with SLR14 (two mice) or vehicle (three mice). 24 h after the third treatment, tumors were harvested, and total RNAs were extracted for RNA-seq. **(A)** Volcano plot of differentially expressed genes between SLR14-treated versus vehicle-treated tumors. **(B)** Heat map of differentially expressed genes involved in type-I IFN-stimulated gene (ISG) between SLR14-treated versus vehicle-treated tumors. Data were generated from one experiment.

To explore the potential mechanisms involved in in vivo antitumor effect of SLR14, we first performed a transcriptomic analysis of YMR1.7 tumors 24 h after the third cycle of i.t. treatment of SLR14 or vehicle (from day 7 to day 13, every 3 d) by RNA sequencing (RNA-seq). As expected from our previous study (Linehan et al., 2018), most of genes associated with the RIG-I pathway, type-I IFN, and IFN-stimulated gene were significantly up-regulated after SLR14 treatment (Figs. S3 A and 4, A and B). Further analysis showed that many genes associated with lymphocyte activation and differentiation, antigen presentation (e.g., MHC I genes including H2-Q6, H2-Q7, H2-Q5, H2-K1, and H2-K2), cytokines/chemokines and their receptors (Ifnb1, Tnf, Il-1, Il-18, Nos2, Cxcl10, Cxcl5, etc.) were also significantly up-regulated (Fig. S3, B and C; and Fig. 4 A). While Cd274 (PD-L1) was significantly up-regulated, we did not observe any significant changes in lymphocyte exhaustion genes including Pdcd1 (PD-1), Ctla4 (CTLA4), Tigit, Lag3, etc. (Fig. S3 D). Moreover, gene ontology (GO) analysis showed that many genes associated with immune activation or defense were significantly up-regulated (Fig. S3, E and F).

**Figure 5.**
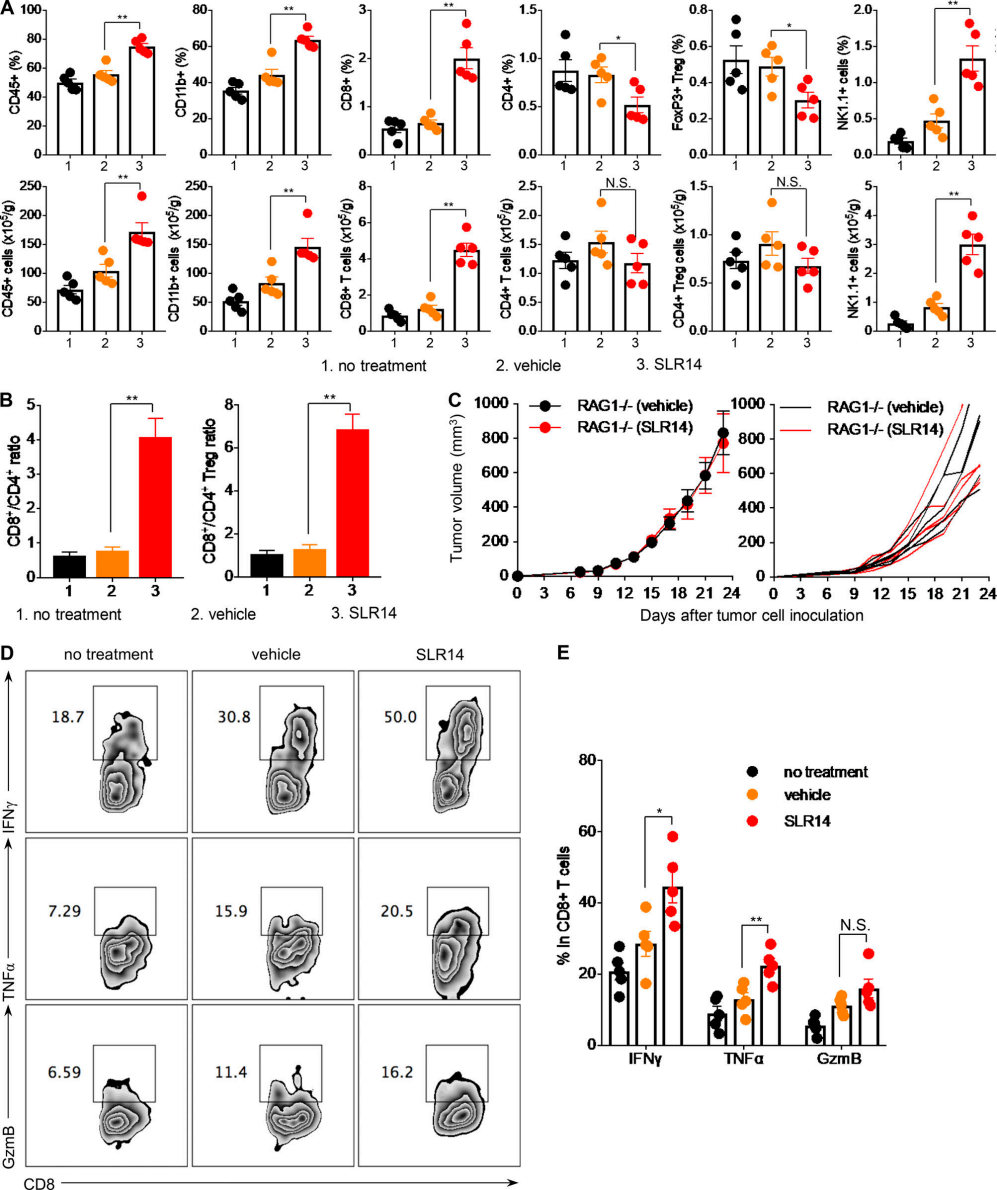
**I.t. SLR14 delivery enhances tumor infiltration of cytotoxic T lymphocytes and myeloid cells.** Subcutaneous YMR1.7 melanoma was established in C57BL/6J mice and i.t. treated with vehicle, SLR14, or no treatment. 3 d after the fifth treatment, tumors were harvested and digested with 0.5 mg/ml Collagenase D and 40 µg/ml DNase I. Single-cell suspensions were prepared for flow cytometry analysis. **(A)** Percentages (top) and quantities (bottom) of tumor-infiltrating CD45^+^, CD11b^+^, CD8^+^, CD4^+^, FoxP3^+^CD4^+^, or NK1.1^+^ cells in each group. All T cells were CD44^+^. The cell numbers were normalized based on the tumor weight. Error bars = SD. 1, no treatment; 2, vehicle; 3, SLR14. **(B)** The ratio of tumor-infiltrating CD8^+^ T cells/CD4^+^ T cells or CD8^+^ T cells/CD4^+^FoxP3^+^ T reg cells in each group. Error bars = SD. **(C)** Subcutaneous YMR1.7 melanoma growth in RAG1^−/−^ mice treated with vehicle or SLR14. Treatment protocol was the same as described in Fig. 1 A. Left: Tumor growth curves (error bars = SD) for each group of mice. Right: Tumor growth curves of individual mice in each group. **(D and E)** IFNγ, TNFα, and GzmB productions of tumor-infiltrating CD8^+^ T lymphocytes after i.t. treatment (error bars = SD). Five mice per group. Unpaired *t* test was used for statistical analysis. *, P < 0.05; **, P < 0.01. N.S., no significance. Results are representative of two independent experiments.

Next, we evaluated the immune infiltrates in tumor 3 d after the fifth cycle of i.t. treatment of SLR14, vehicle, or no treatment by flow cytometry. Compared with the controls (vehicle or no treatment), SLR14 treatment induced a significant increase of tumor-infiltrating CD45^+^ leukocytes, including CD11b^+^ myeloid cells, CD8^+^ T cells, and NK1.1^+^ cells, whereas CD4^+^ cells or CD4^+^FoxP3^+^ T reg cells were significantly decreased (Fig. 5 A, top). Here all tumor-infiltrating CD4^+^ or CD8^+^ T cells were CD44^+^, indicating that they were activated or possibly antigen-experienced. We observed a four- to sixfold increase of CD8^+^/CD4^+^ or CD8^+^/CD4^+^ T reg ratios in SLR14-treated mice compared with control groups (Fig. 5 B).

As the ratio of CD8^+^ to CD4^+^FoxP3^+^ T regs in the tumor microenvironment is highly associated with the T cell–based immune response against tumor after treatment, we speculated that tumor-infiltrating T cells, including both CD8^+^ and CD4^+^ T reg cells, played an important role in SLR14-driven antitumor immunity. To test this, we established YMR1.7 tumor in RAG1^−/−^ mice (lack of T cells and B cells) and i.t. treated them with SLR14 or vehicle. We did not observe any significant difference in tumor growth between SLR14-treated and vehicle-treated mice (Fig. 5 C), indicating that adaptive immune response is required for the clearance of YMR1.7 tumor following SLR14 treatment. Cytokine production assay also showed that, compared with controls (vehicle or no treatment), SLR14 treatment promoted tumor-infiltrating CD8^+^ T lymphocytes to produce much higher levels of IFNγ and TNFα (Fig. 5, D and E). In addition, we observed a slight increase of CD8^+^ T lymphocytes in dLN after SLR14 i.t. treatment, while their TNFα and granzyme B (GzmB) productions were significantly increased, compared with controls (vehicle or no treatment; Fig. S4, A–C). These findings indicate that SLR14 i.t. treatment also induces the activation of cytotoxic T lymphocytes in dLN.

The antitumor effect of i.t. injection of CpG has been reported to be dependent on IL-12 (Yin et al., 2016). To address whether SLR14 depends on IL-12 in its in vivo antitumor activity, we injected neutralizing anti–IL-12 antibody together with SLR14 or CpG i.t. injection in YMR1.7 tumor (Fig. S5 A). To normalize for the effect of the carrier, this time, both SLR14 and CpG were formulated in jetPEI before injection. We did not observe any significant change in tumor growth in SLR14 and anti–IL-12 cotreated mice, while in CpG and anti–IL-12 cotreated mice, a slight increase of tumor growth was detected (Fig. S5, B and C). Accordingly, poor survival was observed in CpG and anti–IL-12 cotreated mice, whereas SLR14 and anti–IL-12 cotreated mice did not exhibit any significant change in survival (Fig. S5 D). These data demonstrate that, unlike CpG, SLR14-induced antitumor immunity in vivo is not mediated by cytokine IL-12.

### SLR14 exhibits robust antitumor effect in B16 melanoma

**Figure 6.**
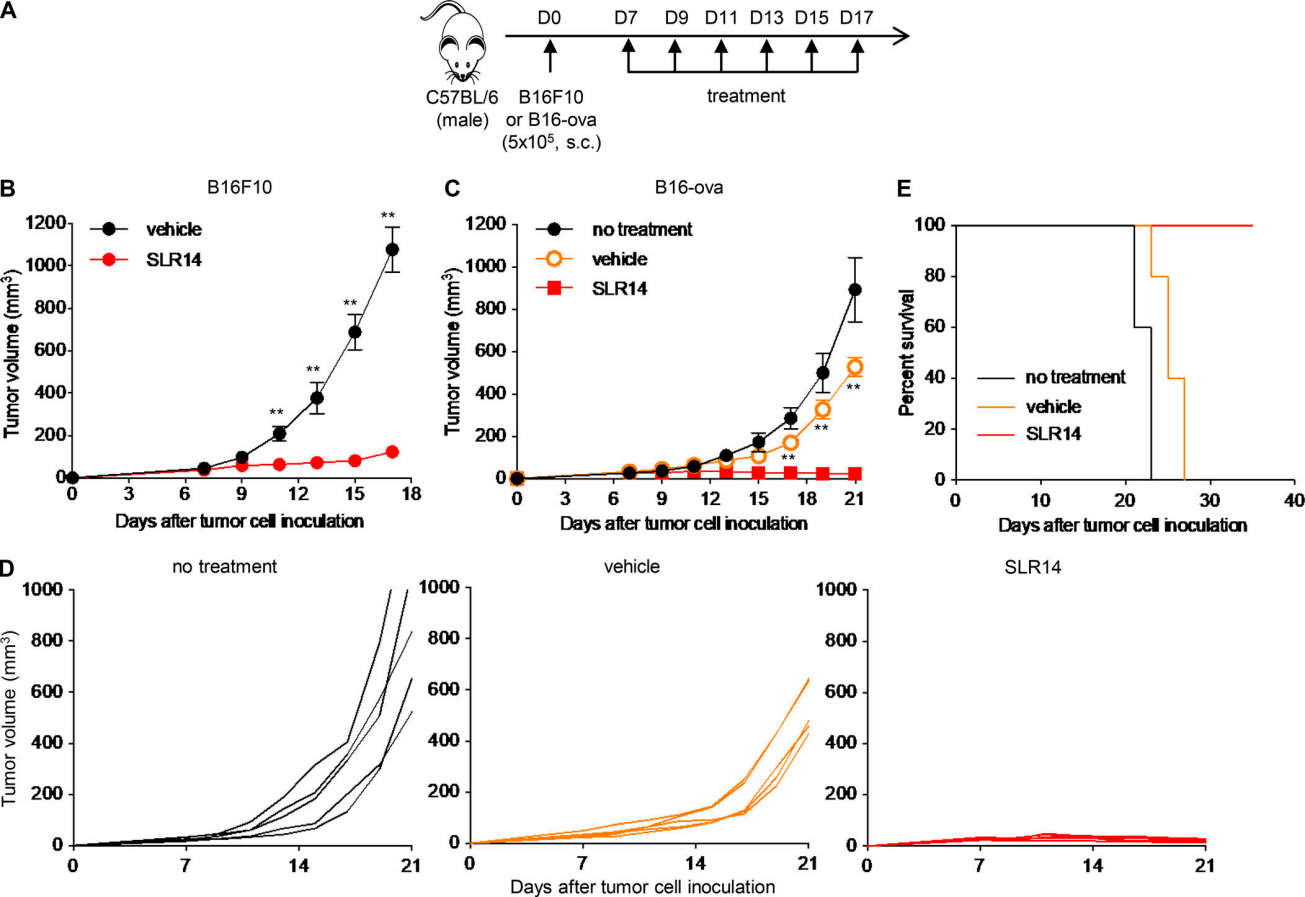
**SLR14 exhibits robust antitumor effect in B16 melanoma. (A)** Subcutaneous B16F10 or B16-ova model was established in C57BL/6 mice. At day 7 after injection, the mice with similar tumor volumes received no treatment or were i.t. treated with 25 µg SLR14 or vehicle. Treatment protocol was the same as that used in YMR1.7 or MC38 model. **(B)** Average tumor volume of B16F10-bearing mice (error bars = SD). **(C)** Average tumor volume of B16-ova–bearing mice (error bars = SD). Significance (**) was found between vehicle and SLR14 groups. **(D)** Tumor growth curves of individual B16-ova–bearing mice after treatment. **(E)** Survival curve of B16-ova–bearing mice after treatment. Five mice per group. Multivariate analysis of variance and multiple *t* test was used for statistical analysis. **, P < 0.01. Results are representative of at least two independent experiments.

In the above experiments, we used immunogenic cancers YMR1.7 and MC38 to evaluate antitumor efficacy of SLR14 in vivo. To assess the broad antitumor effect of SLR14, we used the poorly immunogenic melanoma cell line B16F10, which is resistant to traditional immune therapies including immune checkpoint inhibitors, or B16F10 expressing ovalbumin (B16-ova), to establish subcutaneous melanoma models in C57BL/6J mice (Fig. 6 A). We found that B16F10 growth in SLR14-treated mice was dramatically inhibited, whereas the vehicle-treated tumors grew rapidly (Fig. 6 B). The antitumor effect on B16-ova model was even more striking: after six cycles of i.t. treatment with SLR14, tumors failed to grow and eventually completely disappeared (Fig. 6, C and D). The tumor growth inhibition in SLR14-treated mice lasted for at least 12 d after last treatment, when all tumors in the control mice already reached the euthanasia criterion (tumor volume >1 cm^3^). Accordingly, these SLR14-treated mice displayed a significantly long-term survival (Fig. 6 E). These findings indicate that SLR14 induces a robust antitumor activity in the poorly immunogenic B16 melanoma model.

### Antitumor effect of SLR14 in B16 melanoma relies on both T cells and non-T cells

**Figure 7.**
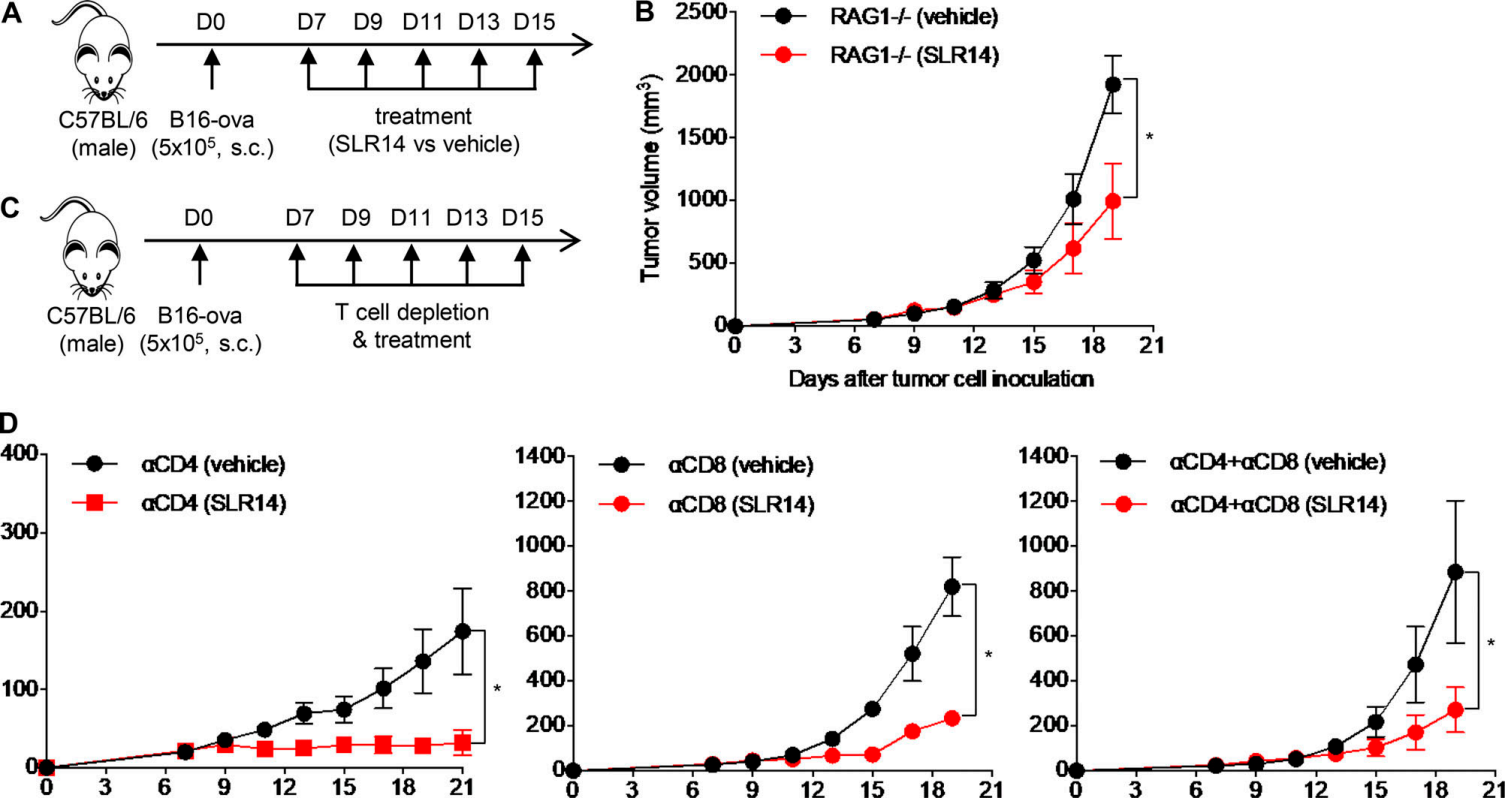
**Antitumor effect of SLR14 in B16-ova melanoma is partially mediated by T cells. (A)** Subcutaneous B16-ova melanoma model was established in RAG1^−/−^ mice. At day 7 after injection, the mice with similar tumor volumes were i.t. treated with SLR14 or vehicle (five mice per group). The treatment protocol was the same as described in Fig. 6. **(B)** Average tumor volume for each group of mice (error bars = SD). **(C)** Subcutaneous B16-ova melanoma model was established in C57BL/6J mice. At day 7 after injection, the mice with similar tumor volumes were i.p. injected with T cell depletion antibodies (anti-CD4^+^, anti-CD8^+^, or both anti-CD4^+^ and anti-CD8^+^; five mice per group) at 200 µg/mouse, followed by i.t. injection of SLR14 or vehicle. In vivo T cell depletion was maintained every 3 d. **(D)** Average tumor volume for each group of mice (error bars = SD). Multiple *t* test was used for statistical analysis. *, P < 0.05. Results are representative of two independent experiments.

As SLR14 showed a potent antitumor effect in poorly immunogenic B16F10 or B16-ova melanoma, we wondered whether its antitumor effect still relied on lymphocytes. To address this question, we first generated subcutaneous B16-ova melanoma in RAG1^−/−^ mice and i.t. treated them with the same dose of SLR14 or vehicle as for YMR1.7 (Fig. 7 A). Compared with the vehicle-treated tumors, the SLR14-treated tumors showed a significant delay of tumor growth after five cycles of treatment even in RAG1^−/−^ mice (Fig. 7 B). This was different from what we observed in YMR1.7 model (Fig. 5 C), indicating a lymphocyte-independent component of SLR14-induced antitumor response against B16-ova. To further probe this phenotype, we established subcutaneous B16-ova melanoma in WT mice. Starting from day 7 after injection, we performed in vivo T cell depletion (CD4^+^, CD8^+^, or both CD4^+^ and CD8^+^) followed with i.t. treatment of SLR14 or vehicle for a total of five doses (Fig. 7 C). Compared with vehicle-treated CD8^+^ T cell–depleted mice, SLR14-treated CD8^+^ T cell–depleted mice showed a significant tumor growth delay, indicating a CD8^+^ T cell–independent antitumor mechanism. We also found that the tumors in CD4^+^ T cell–depleted mice grew very poorly, with or without SLR14 treatment, likely reflecting T reg depletion leading to tumor control (Fig. 7 D). Collectively, these results demonstrate that both T cells and non-T cells are involved in SLR14-induced antitumor response against B16-ova melanoma.

### I.t. SLR14 delivery induces an effective abscopal effect on untreated distant tumors

**Figure 8.**
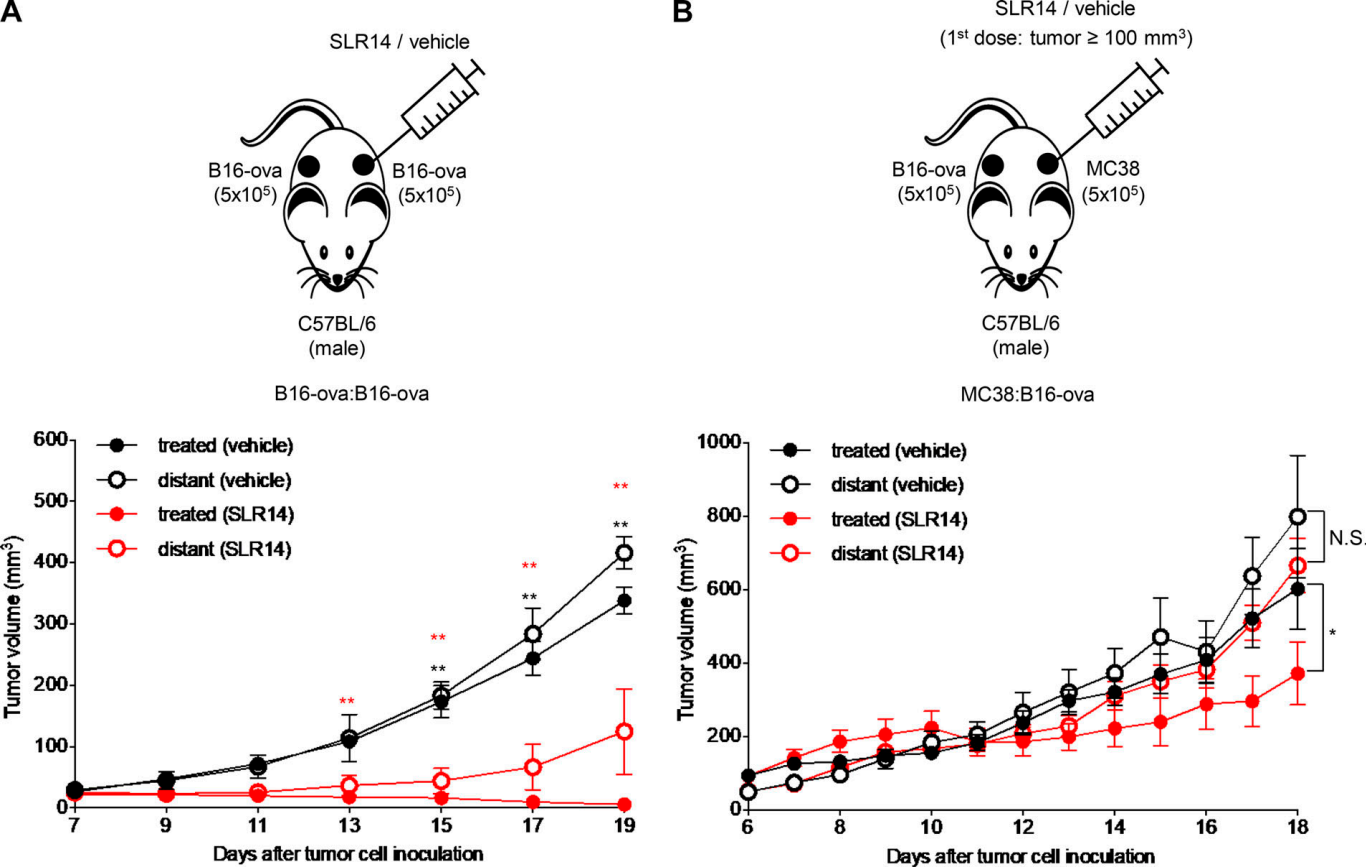
**SLR14 i.t. treatment induces an effective abscopal effect. (A)** Bilateral B16-ova:B16-ova tumor model was established in both flank sides of C57BL/6J mice. At day 7 after injection, only one side of tumor was i.t. treated with SLR14 or vehicle (five mice per group). Treatment protocol was the same as described in Fig. 6. Tumor growth of both flank sides was monitored every 2 d. The average tumor volume (error bars = SD) of B16-ova at both treated and untreated (distant) flank sides is shown. Multivariate analysis of variance was used for statistical analysis. **, P < 0.01. Red double asterisks indicate comparison between treated (vehicle) and treated (SLR14). Black double asterisks indicate comparison between distant (vehicle) and distant (SLR14). **(B)** Bilateral MC38:B16-ova tumor model was established in both flank sides of C57BL/6J mice. Only MC38 tumor was treated with SLR14 or vehicle (five mice per group) when MC38 volume reached 100 mm^3^ (day 10–11). The treatment protocol was the same as described in Fig. 6. Tumor growth of both flank sides was monitored every day. The average tumor volume (error bars = SD) of MC38 or B16-ova at both treated and untreated (distant) flank sides is shown. Multiple *t* test was used for statistical analysis. *, P < 0.05. N.S., no significance. Results are representative of two independent experiments.

Given that SLR14 could target many cells, including T and non-T cells, to induce robust antitumor responses against the SLR14-treated B16-ova, we speculated that it might also induce a systemic antitumor immune response (abscopal effect) in B16-ova-bearing mice. To investigate this possibility, we established bilateral B16-ova:B16-ova subcutaneous tumors in C57BL/6J mice by transplanting tumor cells into the right and left flanks. At day 7 after tumor injection, the mice bearing two B16-ova tumors with similar sizes were selected, and only the right flank tumors were i.t. treated with SLR14 or vehicle (Fig. 8 A). Compared with the right flank B16-ova tumors treated with vehicle, the right flank B16-ova tumors treated with SLR14 were significantly inhibited. This observation is consistent with the results shown in Fig. 6. In SLR14-treated mice, the left flank B16-ova tumors that did not receive SLR14 treatment were still larger than the SLR-treated right flank B16-ova tumors. However, they were still significantly inhibited compared with the untreated left flank B16-ova tumors in vehicle-treated mice (Fig. 8 A). To test if the abscopal effect would affect tumor growth of an unrelated tumor, we generated an MC38:B16-ova bilateral model and started SLR14 or vehicle i.t. treatment only in MC38, which was injected into the right flank. SLR14-treated MC38 tumors were significantly inhibited relative to vehicle-treated MC38, and no significant effect on distant (untreated) B16-ova tumors growing in the left flank was detected (Fig. 8 B). Taken together, these results demonstrate that i.t. delivery of SLR14 can induce an effective abscopal effect on an untreated distant tumor when they are of the same type.

### I.t. SLR14 treatment significantly impedes tumor metastasis

**Figure 9.**
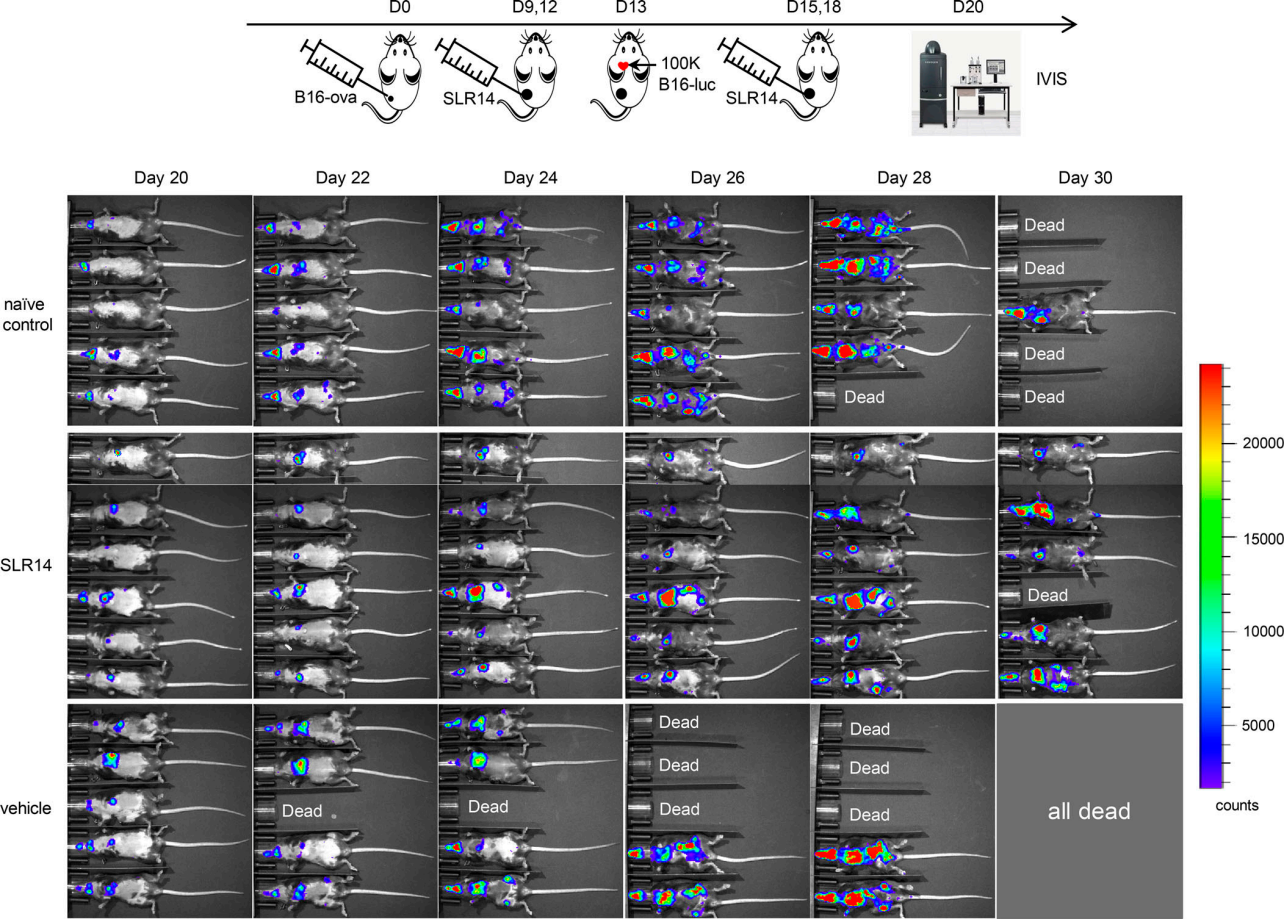
**I.t. SLR14 treatment significantly impedes tumor metastasis.** Subcutaneous B16-ova melanoma model was established in C57BL/6J mice. At day 9 after injection (tumor volume ≥100 mm^3^), the mice were i.t. treated with 1 mg/kg (25 µg) SLR14 or vehicle. The treatment was performed every 3 d for a total of four doses. 24 h after the second i.t. treatment (D13), 10^5^ B16-Fluc cells were injected into the left ventricle of SLR14- or vehicle-treated mice. One group of naive C57BL/6J mice injected with the same numbers of B16-Fluc cells were used as control. Five to six mice per group. 1 wk later (D20), B16-Fluc cells were imaged for bioluminescence at a 10-s exposure setting on the IVIS Spectrum imager. Imaging was performed every other day for 2 wk. Results are representative of two independent experiments.

Our experiments above demonstrated that SLR14 i.t. treatment induced systemic antitumor immune response on a singular distant tumor. We next examined whether SLR14 i.t. treatment was effective in preventing against widely disseminated metastases, as this would be a desirable characteristic of antitumor agent i.t. delivered in a clinical setting. Injection of tumor cells directly into the left ventricle results in systemic metastases and serves as a superior preclinical model for pharmacological intervention (Khanna and Hunter, 2005). We injected luciferase reporter bearing B16F10 (B16-Fluc) cells into the left ventricle of B16-ova–bearing mice after they had received two cycles of i.t. treatment of SLR14 or vehicle. Considering the possible effect of primary tumor on metastatic growth, we also injected B16-Fluc cells into tumor-free naive mice. Bioluminescence imaging was performed 1 wk after B16-Fluc cell injection and at 48-h intervals thereafter. A widespread dissemination and metastatic outgrowth of B16-Fluc tumors in the brain, lung, and other organs was observed in all the injected mice. However, metastatic growth was noticeably decreased in the mice bearing flank tumors treated with SLR14, compared with the signals detected in the vehicle-treated or tumor-free untreated naive control mice. The differences in metastatic burden between treated and control mice increased with time until the eventual death of all mice in the vehicle-treated mice (Fig. 9). These data clearly demonstrate that i.t. SLR14 administration could reduce tumor metastatic burden in the left ventricle injection-based metastasis model.

### B16-cured mice after SLR14 treatment develop immune memory

**Figure 10.**
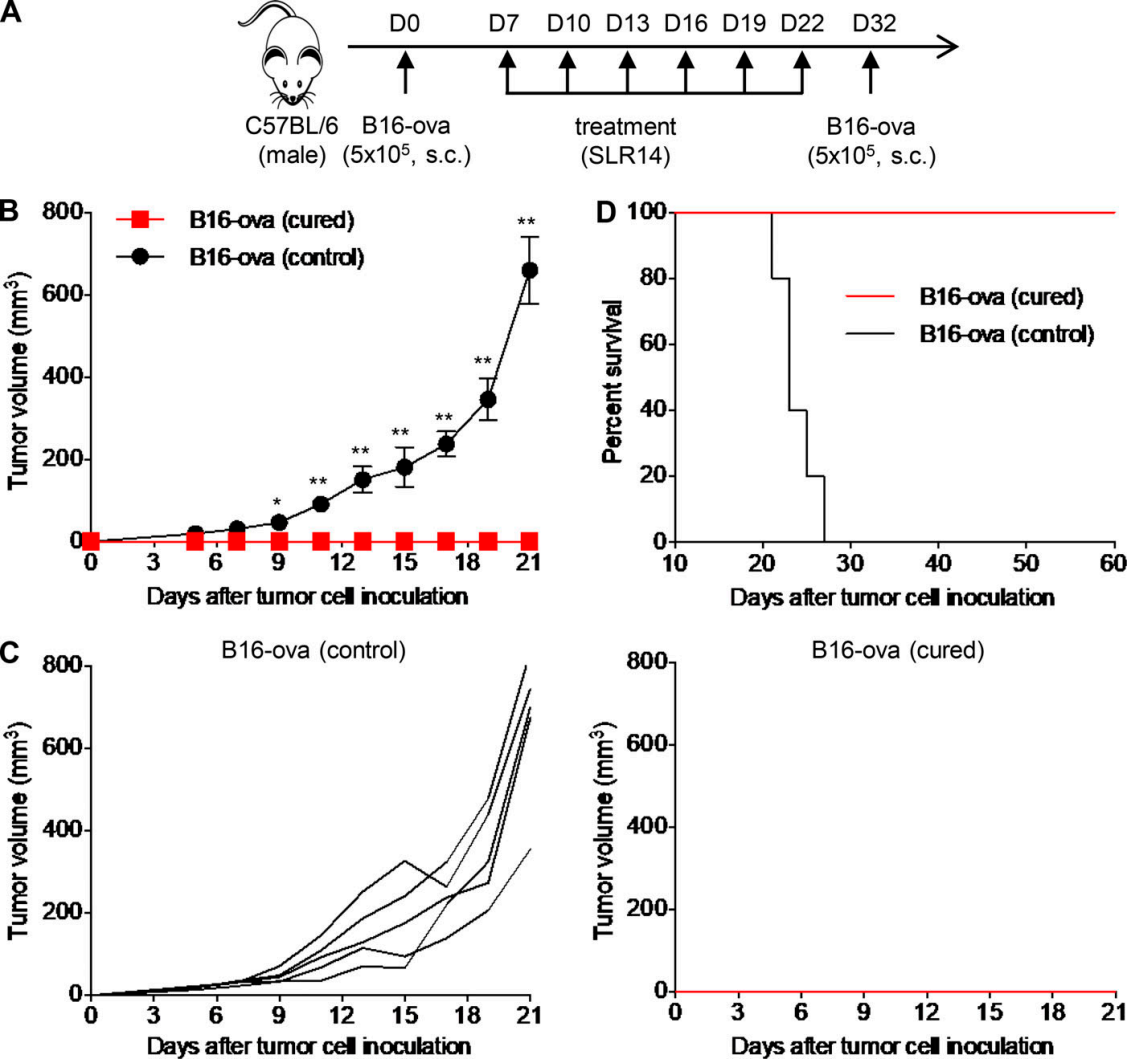
**B16-ova–cured mice after SLR14 treatment develop immune memory. (A)** Subcutaneous B16-ova melanoma model was established in C57BL/6 mice. From day 7 after injection, the mice were i.t. treated with 1 mg/kg (25 µg) SLR14 every 3 d for a total of six doses. 10 d after last treatment, the cured mice (five mice) were challenged with B16-ova at the cured flank side. Age- and gender-matched naive mice injected with the same numbers of B16-ova were used as controls. Five mice per group. **(B)** Average tumor volume for each group of mice (error bars = SD). Multiple *t* test was used for statistical analysis. *, P < 0.05; **, P < 0.01. **(C)** Tumor growth curves of individual mice in each group. **(D)** Survival curve of tumor-challenged mice. Results are representative of two independent experiments.

In the B16 melanoma experiments above, some tumors were cured by i.t. treatment with SLR14 (Figs. 6, 7, and 8). To test whether this protection is durable and tumor-specific, we first generated B16-ova–cured mice with SLR14 i.t. treatment. At 10 d after last treatment, 5 × 10^5^ B16-ova cells were s.c. injected into these tumor-free mice (Fig. 10 A). In parallel, naive mice challenged with 5 × 10^5^ B16-ova cells were used as controls. Over the next 3 wk after tumor challenge, we did not observe any tumor growth in B16-ova–cured mice, while naive mice developed large tumor growth and died within 27 d (Fig. 10, B–D). These findings suggest that tumor-cured mice after i.t. treatment with SLR14 develop immune memory to tumor challenge.

## Discussion

The suppressive tumor microenvironment is a critical barrier for the success of cancer immunotherapy. Based on the infiltration of T cells, tumor can be divided into immunogenic or nonimmunogenic types (Gajewski et al., 2017). Currently, immune checkpoint blockade (ICB) therapy has shown durable clinical benefit, but its efficacy is limited to a minority of cancer patients whose tumors are immunogenic and preinfiltrated by T cells (Binnewies et al., 2018). In this study, our data reveal that, after i.t. administration, SLR14 induces an effective antitumor response in both immunogenic and nonimmunogenic tumors. In immunogenic tumors, SLR14 significantly increases the tumor infiltration of cytotoxic CD8^+^ T cells, NK cells, and CD11b^+^ myeloid cells while decreasing the immunosuppressive CD4^+^FoxP3^+^ T reg cells. Transcriptomic analyses further revealed a significant up-regulation of many genes associated with immune defense including lymphocyte activation, cytokines, chemokines, and antigen presentation after SLR14 i.t. treatment, which is consistent with the increased antitumor immune infiltrates and tumor clearance. These data suggest that SLR14 profoundly changes the proportions of cytotoxic lymphocytes over immunosuppressive cells in the tumor microenvironment, leading to a strong antitumor immune response. In addition, our results from combination therapy experiments with anti-PD1 indicate that SLR14 can serve as a powerful immune adjuvant to enhance the efficacy of ICB immunotherapy.

It has been reported that poorly immunogenic or nonimmunogenic tumors rarely respond to ICB due to a lack of tumor-infiltrating lymphocytes (Binnewies et al., 2018). Surprisingly, SLR14 showed a remarkable antitumor efficacy in a poorly immunogenic tumor: B16 melanoma. Most B16-ova melanoma could be cured by SLR14 i.t. injections, and these cured mice generated an antigen-specific memory response against the tumor. Our results also suggest that SLR14 i.t. treatment changes the local tumor microenvironment, converting poorly immunogenic tumor into immunogenic tumor, and inducing a durable antitumor immunity. Although further investigation of the mechanisms for antitumor effects of SLR14 in nonimmunogenic tumor is required, our data indicate that SLR14 monotherapy elicits both T cell–mediated and non–T cell–mediated responses resulting in tumor cell death.

Previous studies have shown that treatment with RIG-I agonists induces direct cancer cell death or cancer immunogenic cell death that is mainly mediated by immune cells, including DCs, NK cells, and CD8^+^ T cells (Poeck et al., 2008; Besch et al., 2009; Chiappinelli et al., 2015; Roulois et al., 2015; Yu et al., 2016). Our data show that SLR14 is mainly taken up by CD11b^+^ myeloid cells, which could include Ly6G^+^ tumor-associated neutrophils, tumor-associated macrophages, and tumor-associated DCs (including Ly6C^+^ inflammatory DC subset). In addition, our data also indicate that the CD8^+^ T cell–mediated immune response is well correlated with the antitumor efficacy of SLR14. Whether and how RIG-I activation in tumor-associated myeloid cells regulates antitumor T cell response is an interesting question that remains to be addressed in the future. In addition, tumor cells also likely take up SLR14, as there are ∼20% CD45^−^ cells showing SLR14 uptake. This suggests that SLR14 may induce direct tumor cell apoptosis. In poorly immunogenic tumors, the tumor cell–expressed RIG-I has been reported to be crucial for mediating in vivo therapeutic effect of RIG-I agonists. For example, 5′ ppp-dsRNA i.t. treatment significantly reduces the tumor size of WT B16F10, while it does not reduce the tumor size of RIG-I KO B16F10 in vivo (Engel et al., 2017). It was also recently reported that SLR20, an SLR family member that is 20 bp in length, directly targets 4T1 breast cancer cells, which are another poorly immunogenic class of tumors, to activate the RIG-I signaling pathway (Elion et al., 2018). While we did not investigate the target cells in which SLR14 induces RIG-I activation, our data from RAG1^−/−^ mice indicate a small but significant T cell–independent antitumor activity elicited by SLR14, presumably representing direct tumor cell death induced by SLR14 or induction of myeloid cell–dependent removal of the tumor. As RIG-I is ubiquitously expressed in all cells including tumor cells, some interesting questions arise: are there any specific targets for RIG-I agonists in vivo when the tumor immunophenotype is different? Which cell population (e.g., tumor cells or nontumor cells) targeted by RIG-I agonists in vivo can induce a potent antitumor response? It has been reported that malignant cells are highly sensitive to a RIG-I proapoptotic signaling pathway, whereas normal cells do not succumb to apoptosis as they up-regulate antiapoptotic protein Bcl-xL (Besch et al., 2009). This can be leveraged to treat metastatic tumors with SLR14.

Abscopal effect is a systemic response induced by local treatment (Ngwa et al., 2018). In this study, we found that SLR14 i.t. treatment induced an increase of cytotoxic CD8^+^ T lymphocytes not only in tumor but also in the dLN, and an increase in CD11b^+^ cells containing SLR14 in the dLN (but not in the ndLN). These data are consistent with an enhanced priming of tumor-specific CD8^+^ T cells in the dLN following SLR14 i.t. injection, therefore inducing a systemic antitumor immune response. Indeed, both nonimmunogenic (B16) and immunogenic (MC38) bilateral tumor models showed an abscopal effect with SLR14. Using the bilateral MC38/B16-ova model, we demonstrated that the SLR14-induced abscopal effect was tumor specific. Additionally, the B16-Fluc metastasis model in SLR14-treated mice further indicates a possible systemic antitumor immune response induced by SLR14 i.t. treatment. Abscopal effect has been thought to be mainly mediated by cytotoxic T lymphocytes (Rodríguez-Ruiz et al., 2018). Whether CD8^+^ T lymphocytes or other unknown mechanisms are involved in SLR14-induced abscopal effect needs to be further investigated.

In summary, our results demonstrate that SLR14, a synthetic RIG-I agonist, induces a potent in vivo antitumor effect in immunogenic or poorly immunogenic cancers by activating the cytosolic RIG-I signaling pathway in different cell populations. Compared with other PRR agonists, such as CpG, used as control in this study, SLR14-induced antitumor effect is not IL-12 dependent. Our findings in this study suggest that SLR14 is a promising therapeutic RIG-I agonist for a broad spectrum of cancer types. We believe a better understanding of RIG-I activation in the tumor microenvironment may yield novel approaches for the next generation of cancer immunotherapy.

## Materials and methods

### Mice and tumor cells

C57BL/6J and C57BL/6J RAG1^−/−^ mice were purchased from the Jackson Laboratory, bred, and housed in pathogen-free conditions at the animal facility of Yale Animal Resources Center. Approximately 8–12-wk-old male mice (∼25 g per mouse) were used for experiments. All procedures were performed under the protocols approved by the Yale Institutional Animal Care and Use Committee.

Five mouse tumor cell lines were used in this study: B16F10, B16-ova, B16F10-luciferase (B16-Fluc) melanoma cells, and MC38 colon cancer cells were cultured in DMEM with 10% FBS and 1% antibiotics, and the melanoma cell line YMR 1.7 (Wang et al., 2017b) were maintained in DMEM/F12 media containing 10% FBS, 1% nonessential amino acids, and 1% penicillin-streptomycin.

### Synthesis, purification, and labeling of the SLR-14 oligonucleotide

The triphosphorylated RNA oligonucleotides SLR-14 (5′-pppGGAUCGAUCGAUCGUUCGCGAUCGAUCGAUCC-3′) and SLR-14-amino (5′-pppGGAUCGAUCGAUCGUXCGCGAUCGAUCGAUCC-3′, where X = aminomodifier C6dT; Glen Research), were prepared essentially as described (Mihaylova et al., 2018). Briefly, removal of the oligonucleotide from the polymer support and base deprotection was performed in a 1:1 mixture of 40% methylamine (Sigma-Aldrich) and 30% ammonium hydroxide (JT Baker) at 65°C for 15 min. The solution was cooled on ice for 10 min, transferred to a new vial, and evaporated to dryness. 500 µl of absolute ethanol was added, and the mixture was evaporated to dryness again. To deprotect the 2′-OH groups, the dry oligonucleotide was incubated with 500 µl of a 1 M solution of tetrabutylammonium fluoride in tetrahydrofuran (Sigma-Aldrich) at room temperature for 36 h. 500 µl of 2 M sodium acetate (pH 6.0) was added, and the solution was evaporated to a 500–600 µl volume, extracted with 3× 800 µl of ethyl acetate, and ethanol precipitated. The RNA oligonucleotide was then purified on a 16% denaturing polyacrylamide gel.

For fluorescent labeling, the purified SLR-14-amino oligonucleotide was dissolved in 200 µl of 0.25 M sodium bicarbonate buffer (pH 9.2). Then, a solution containing 0.5 mg of Alexa Fluor 647 NHS ester (Life Technologies Corp.) in 200 µl *N*,*N*-dimethylformamide was added, and the reaction mixture was incubated at room temperature for 2 h. The labeled oligonucleotide (SLR14-647) was ethanol precipitated and purified on a 20% denaturing polyacrylamide gel.

### In vivo tumor injection and treatment

Either 5 × 10^5^ or 10^6^ tumor cells were subcutaneously injected into the flank of naive syngeneic mice. For bilateral tumor model, both right and left flanks were injected with equal numbers of the same or different types of tumor cells. When tumor volume reached 40–80 mm^3^ or >100 mm^3^, 1 mg/kg SLR14 was i.t. injected. Briefly, 1 mg/kg (∼25 µg) SLR14 and 4 µl jetPEI (Polyplus Transfection) were diluted and mixed with 5% glucose solution in total 50 µl. After 15 min of incubation at room temperature, a 50-µl complex was carefully injected into the tumor with a 0.5-ml BD Insulin syringe from different directions. When the tumor was small, we injected complex to make a small bubble to cover the whole tumor. I.t. injection was performed every 2–3 d, for a total of 5–6 doses. The tumor-bearing mice with i.t. treatment of vehicle (jetPEI) or water with 5% glucose (no treatment) were used as controls. In some of the experiments, 25 µg CpG 1826 (CpG) mixed with jetPEI and 5% glucose was used for i.t. injection; 5 or 200 µg anti-PD1 antibody (Bio X Cell) was i.p. injected into the mice; i.t. injection of anti-mouse IL-12 (10 µg per mouse, Bio X Cell) was performed every other day. For in vivo T cell depletion, the mice were i.p. injected with 200 µg anti-mouse CD4 (GK1.5), anti-mouse CD8 (2.43), or both. In vivo T cell depletion was maintained every 3 d.

### RNA extraction, library preparation, and sequencing

Total RNAs were extracted, and the quality was determined by estimating the A260/A280 and A260/A230 ratios by nanodrop. RNA integrity was determined by running an Agilent Bioanalyzer gel, which measures the ratio of the ribosomal peaks.

#### RNA-seq library preparation

mRNA was purified from ∼200 ng of total RNA with oligo-dT beads and sheared by incubation at 94°C in the presence of Mg (Kapa mRNA Hyper Prep). Following first-strand synthesis with random primers, second-strand synthesis and A-tailing were performed with dUTP for generating strand-specific sequencing libraries. Adapters with 3′ dTMP overhangs were ligated to library insert fragments. Library amplification was used to amplify fragments carrying the appropriate adapter sequences at both ends. Strands marked with dUTP were not amplified. Indexed libraries that met appropriate cutoffs for the samples passing the size distribution and concentration quality controls were quantified by qRT-PCR using a commercially available kit (KAPA Biosystems), and insert size distribution was determined with the LabChip GX or Agilent Bioanalyzer. Samples with a yield of ≥0.5 ng/µl were used for sequencing.

#### Flow cell preparation and sequencing

Sample concentrations were normalized to 10 nM and loaded onto an Illumina NovaSeq flow cell at a concentration that yields 25 million passing filter clusters per sample. Samples were sequenced using 100-bp paired-end sequencing on an Illumina NovaSeq according to Illumina protocols. The 10-bp dual index was read during additional sequencing reads that automatically follow the completion of read 1. A positive control (prepared bacteriophage Phi X library) provided by Illumina was spiked into every lane at a concentration of 0.3% to monitor sequencing quality in real time.

#### Data analysis

Signal intensities were converted to individual base calls during a run using the system’s Real Time Analysis software. The bulk RNA-seq data were deposited in the Gene Expression Omnibus public database under accession no. GSE136995.

### Tumor digestion and flow cytometry analysis

Tumors were harvested, cut into small pieces with surgical scissors and sharp blade, and then digested in HBSS containing 0.5 mg/ml Collagenase D (Roche) and 40 µg/ml DNase I (Roche) in a 37°C shaker for 20–30 min. Digestion was stopped by adding 0.5 mg/ml EDTA in HBSS, and single-cell suspensions were prepared for antibody staining. The following anti-mouse antibodies obtained from BioLegend were used for the staining and analysis: anti-CD45 (30-F11), anti-CD3 (145-2C11), anti-CD4 (GK1.5), anti-CD8 (53-6.7), anti-CD44 (IM7), anti-FoxP3 (MF-14), anti-NK1.1 (PK136), anti-CD11b (M1/70), anti-IFNγ (XMG1.2), anti-TNFα (TN3-19.12), and anti-GzmB (GB11). Intracellular staining was performed using the eBioscience Intracellular Fixation and Permeabilization Buffer Set (88-8824-00). Dead cells were excluded using 7-aminoactinomycin D staining. The samples were run on a BD LSRII flow cytometer, and data were analyzed using FlowJo software.

### Tumor metastasis model

Primary B16-ova melanomas were established in 8-wk-old C57BL/6J mice as described above. At day 9 after implantation, when the tumor volume reached ∼100 mm^3^, the mice were i.t. treated with 1 mg/kg (25 µg) SLR14 or vehicle. The treatment was performed every 3 d for 4 cycles. 24 h after the second cycle of i.t. treatment, the mice were anesthetized by i.p. administration of ketamine (80 mg/kg) and xylazine (8 mg/kg), followed with an injection of 10^5^ B16-Fluc cells to the left ventricle. As controls, vehicle-treated mice with tumor or naive mice without tumor (*n* = 5) were also injected with B16-Fluc in the left ventricle. At 7 d after left ventricle injection, the mice were retro-orbitally administered with 75 mg/kg D-luciferin and imaged for bioluminescence at a 10-s exposure setting on an IVIS Spectrum imager (PerkinElmer). Imaging was continually performed every other day using the same instrument and exposure parameters for 2 wk.

### Statistical analysis

Data are presented as the mean ± SD. Statistical significance in values between experimental groups was determined by unpaired *t* test or multivariate analysis of variance. P < 0.05 was considered statistically significant (*, P < 0.05; **, P < 0.01).

### Online supplemental material

Fig. S1 shows tumor growth curves of individual YMR1.7- or MC38-bearing mice after SLR14 i.t. treatment. Fig. S2 shows tumor growth curves of individual YMR1.7- or MC38-bearing mice after combination treatment with SLR14 and anti-PD1 antibody. Fig. S3 shows transcriptomic analysis of differentially expressed genes involved in RIG-I pathway, lymphocyte activation and differentiation, cytokines/chemokines and their receptors, and lymphocyte exhaustion, as well as GO analysis of up- or down-regulated genes between SLR14- versus vehicle-treated tumors. Fig. S4 shows the frequency of T cells (CD8^+^, CD4^+^, T reg) in dLNs and their cytokine productions (IFNγ, TNFα, GzmB) after SLR14 i.t. treatment. Fig. S5 shows antitumor efficacy of SLR14 i.t. treatment with or without anti–IL-12 antibody.

## Supplementary Material

Supplemental Materials (PDF)
