# Supplementary material for: Intratumoral delivery of RIG-I agonist SLR14 induces robust antitumor responses
Source: J Exp Med. 2019 Oct 10;216(12):2854–68. doi: 10.1084/jem.20190801 (PMC6888973; doi:10.1084/jem.20190801)
Supplement: Supplemental Materials (PDF) [file JEM_20190801_sm.pdf]

JEM

Supplemental material

Jiang et al., https://doi.org/10.1084/jem.20190801

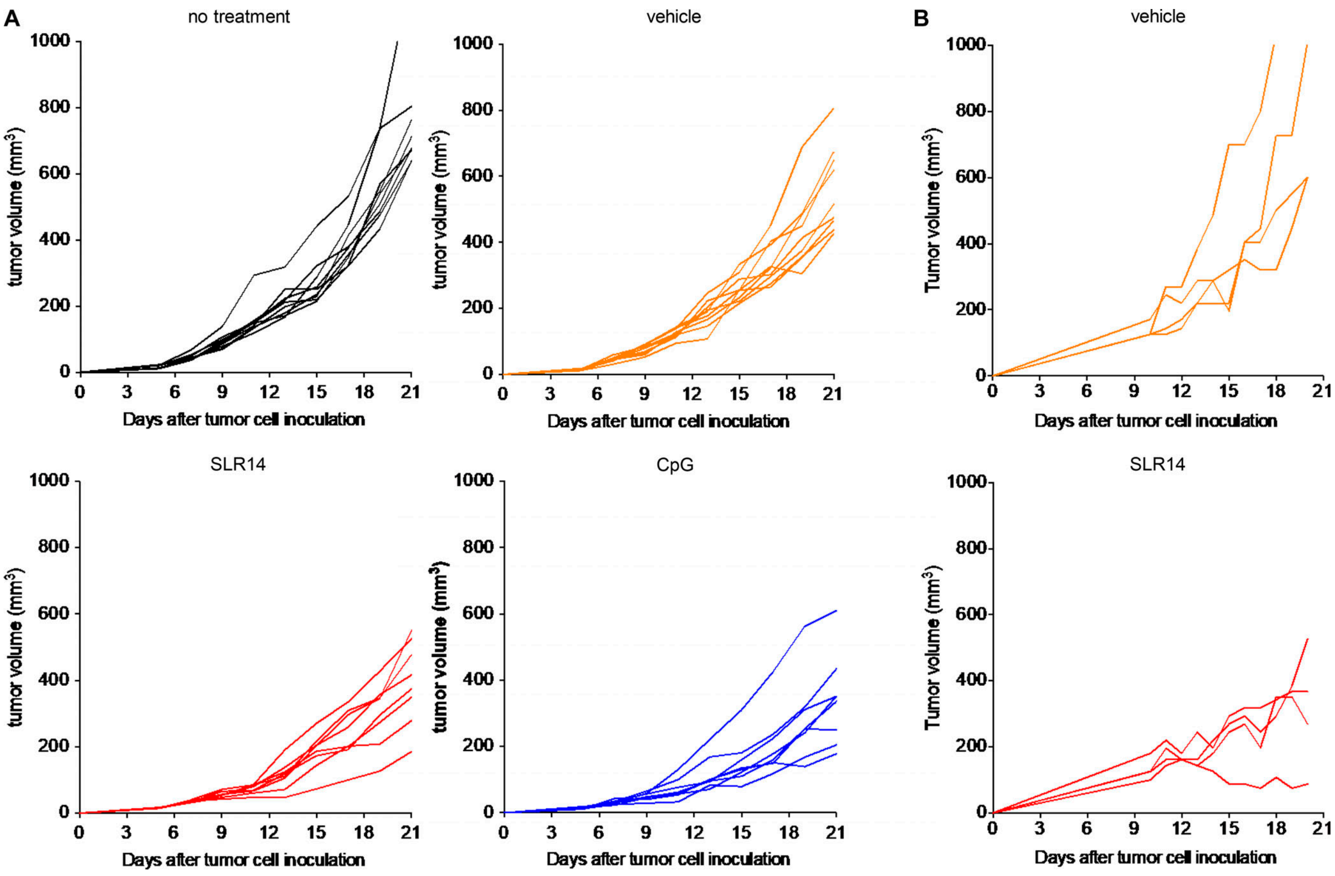

Figure S1. **Tumor growth curves of individual mice after SLR14 i.t. treatment.** Subcutaneous YMR1.7 melanoma or MC38 colon cancer model was established in naive C57BL/6J mice as described in Fig. 1 (A and D), respectively. Tumor-bearing mice were treated with no treatment, vehicle, SLR14, or CpG. The treatment protocol was the same as described in Fig. 1 (A or D). **(A and B)** After treatment, tumor growth of individual YMR1.7-bearing (A, 8–10 mice per group) or MC38-bearing (B, four to five mice per group) mice in each group was monitored. Results are representative of at least two independent experiments.

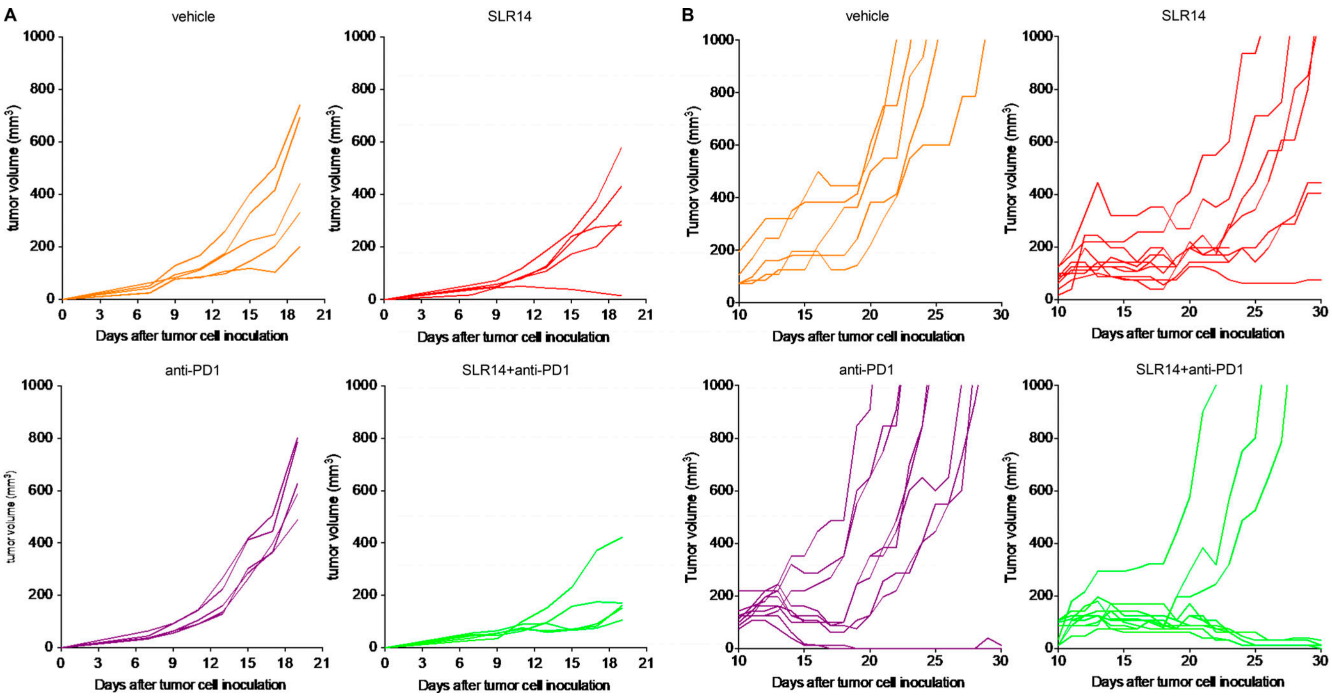

Figure S2. **Tumor growth curves of individual mice after combination treatment with SLR14 and anti-PD1.** Subcutaneous YMR1.7 melanoma or MC38 colon cancer model was established as described in Fig. 2 (A or C), respectively. Tumor-bearing mice were treated with vehicle, SLR14, anti-PD1, or SLR14 and anti-PD1. The treatment protocol was the same as described in Fig. 2 (A or C). **(A and B)** After treatment, the tumor growth of individual YMR1.7-bearing (A, five mice per group) or MC38-bearing (B, 5–10 mice per group) mice in each group was monitored. Results are representative of two independent experiments.

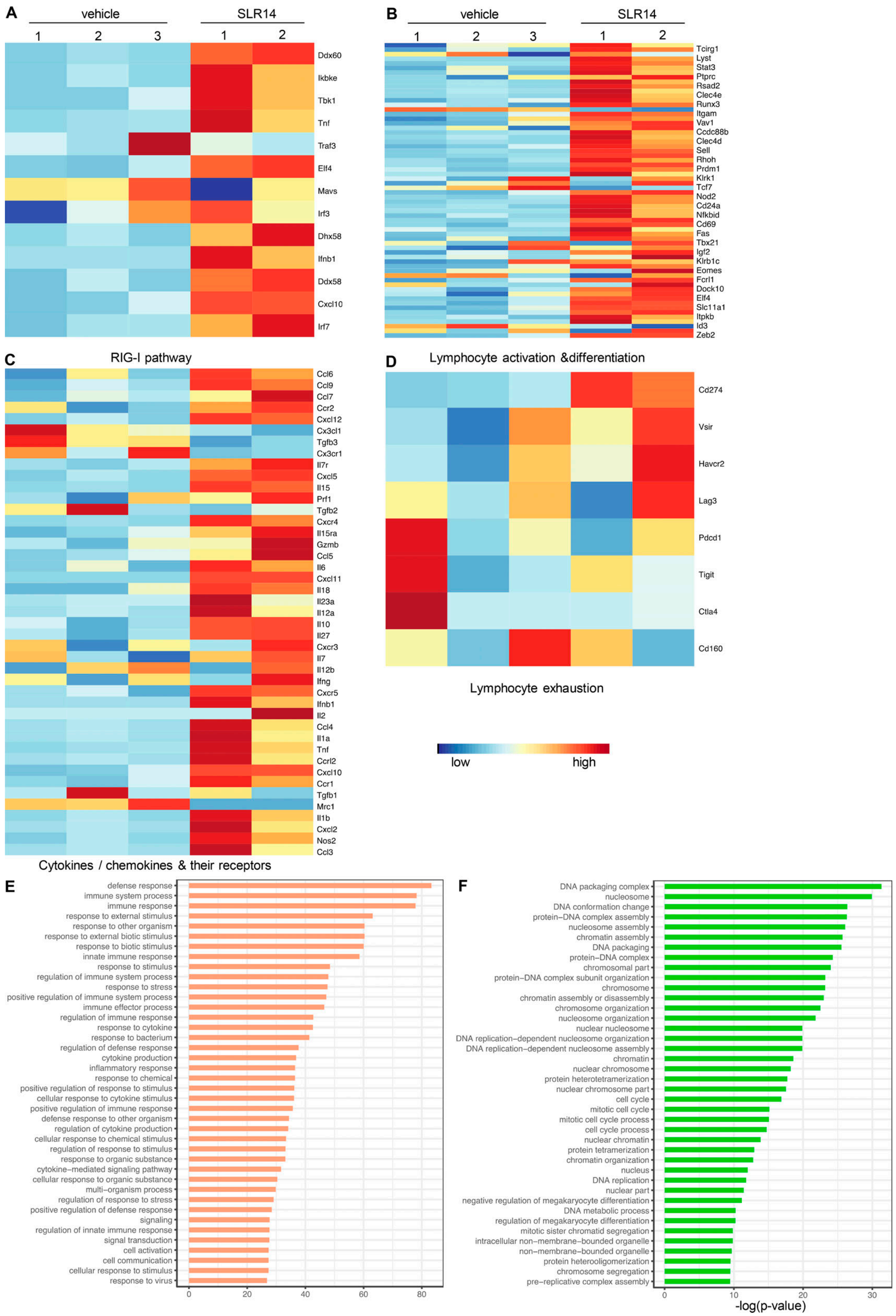

Figure S3. **Transcriptomic analysis of tumor i.t. treated with SLR14 versus vehicle.** Subcutaneous YMR1.7 melanoma model was established in C57BL/6J mice and treated with vehicle (three mice) or SLR14 (two mice). 24 h after the third treatment, tumors were harvested, and total RNAs were extracted for RNA-seq. **(A–D)** Heat maps of differentially expressed genes involved in RIG-I pathway, lymphocyte activation and differentiation, cytokines/chemokines and their receptors, and lymphocyte exhaustion, respectively (SLR14- versus vehicle-treated tumors). GO analysis of differentiated expressed genes between SLR14- versus vehicle-treated tumors was also performed. **(E and F)** The top 40 enriched GO terms of up-regulated (E) and down-regulated (F) genes are listed. Data were generated from one experiment.

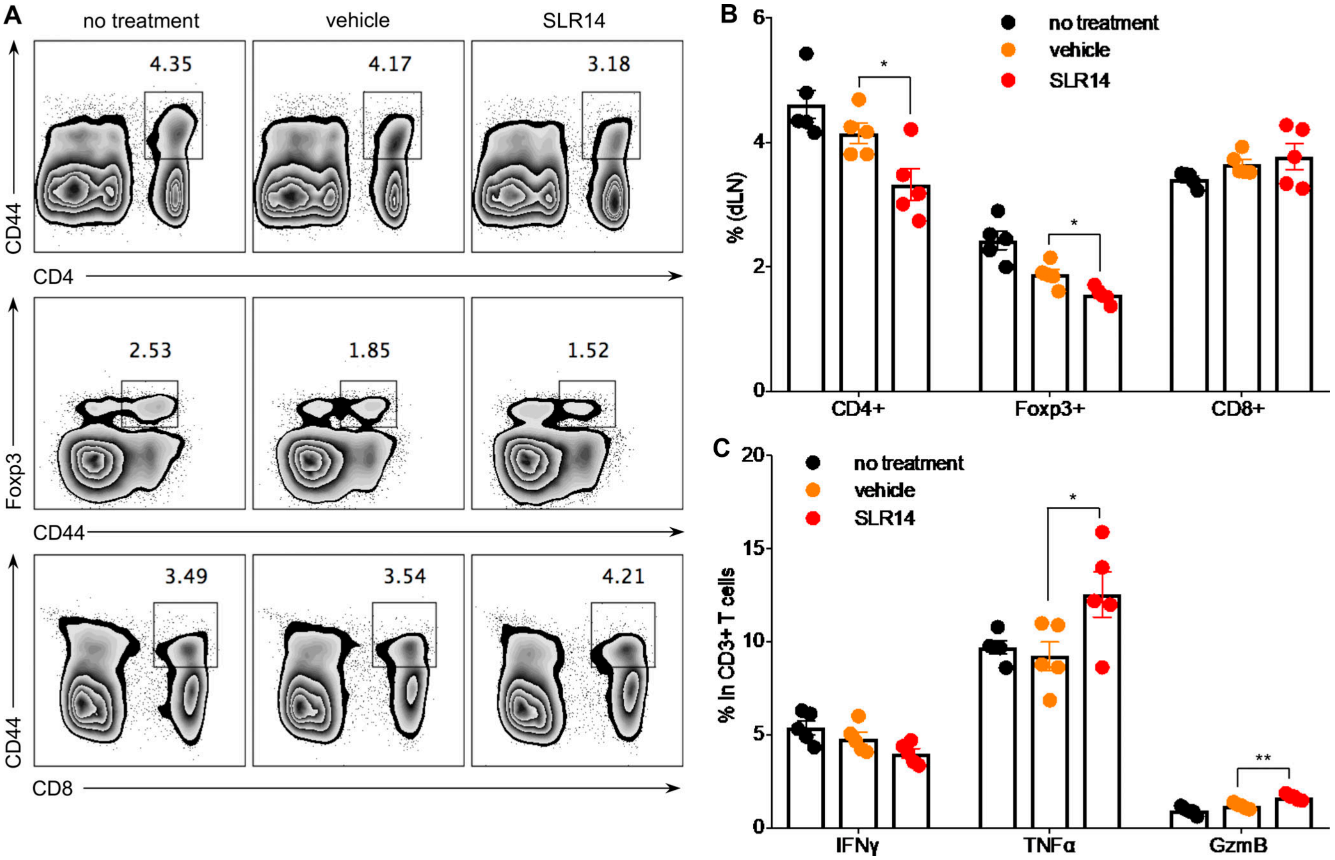

Figure S4. **I.t. treatment of SLR14 induces functional antitumor T cells in dLNs.** Subcutaneous YMR1.7 melanoma model was established in C57BL/6J mice and i.t. treated with vehicle, SLR14, or received no treatment as described in Fig. 5. 3 d after last treatment, tumor dLNs were harvested, and single-cell suspensions were prepared for flow cytometry analysis. **(A and B)** Flow cytometry analysis of $CD44^+CD4^+$ T cells, $CD44^+CD4^+FoxP3^+$ cells, and $CD44^+CD8^+$ T cells in dLNs in each group (error bars = SD). **(C)** IFNγ, TNFα, and GzmB productions of total $CD3^+$ T lymphocytes in dLNs in each group (error bars = SD). Five mice per group. Unpaired *t* test was used for statistical analysis. *, $P < 0.05$; **, $P < 0.01$. Results are representative of two independent experiments.

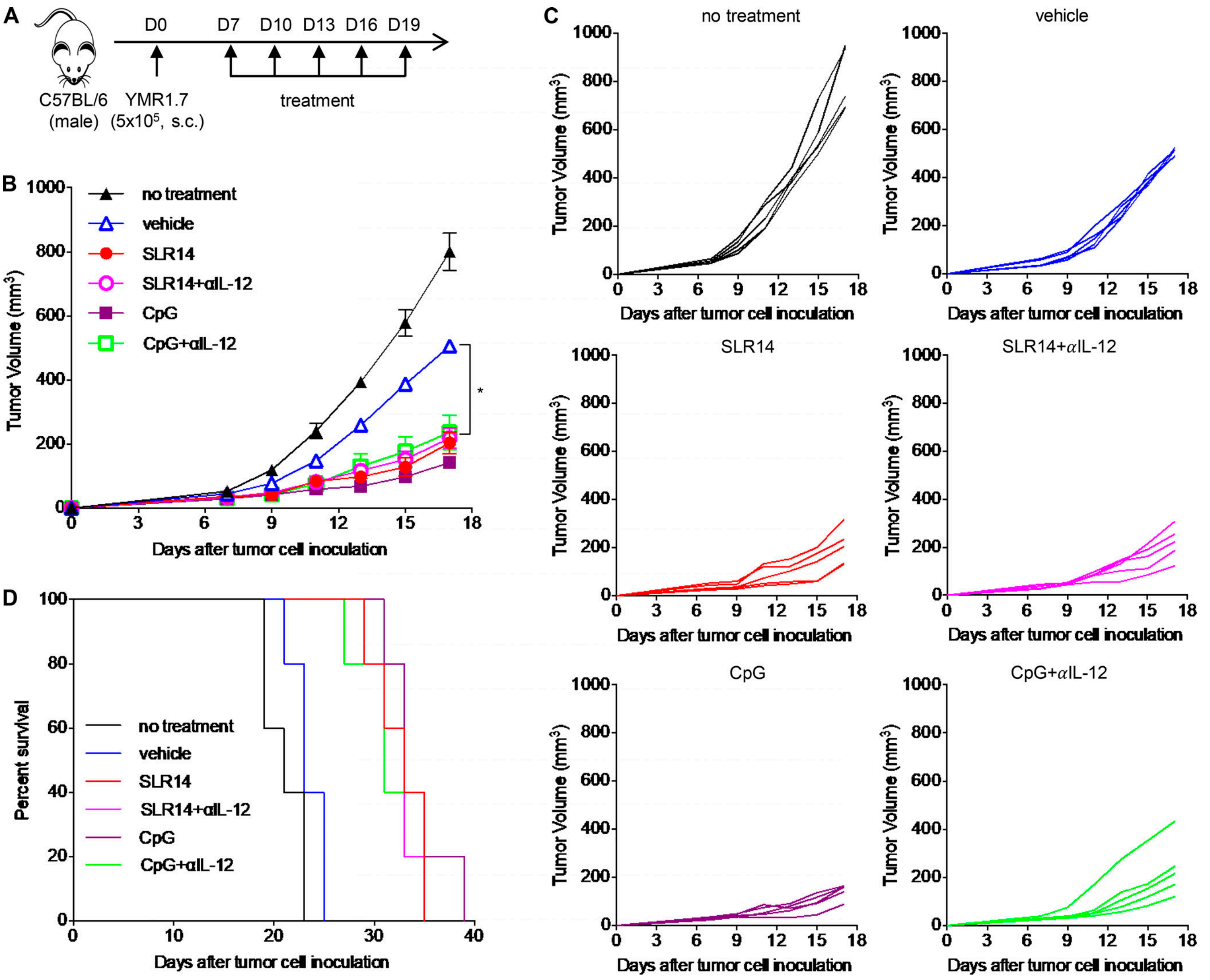

Figure S5. **Antitumor efficacy of SLR14 is not IL-12 dependent.** **(A)** Subcutaneous YMR1.7 melanoma model was established in C57BL/6J mice. At day 7 after injection, the mice with similar tumor volumes were i.t. treated with 1 mg/kg (25 µg) SLR14 or CpG, both formulated with jetPEI, with or without anti–IL-12 (10 µg αIL-12). Some mice i.t. treated with vehicle or no treatment were used as controls. Treatment was performed every 3 d for a total of five doses. **(B)** Average tumor volume for each group of mice (error bars = SD). **(C)** Tumor growth curves of individual mice in each group. **(D)** Survival curve of YMR1.7-bearing mice after treatment. Five mice per group. Multivariate analysis of variance was used for statistical analysis. *, $P < 0.05$. Results are representative of two independent experiments.
